# Activity-based protein profiling reveals deubiquitinase and aldehyde dehydrogenase targets of a cyanopyrrolidine probe†

**DOI:** 10.1039/d1md00218j

**Published:** 2021-08-16

**Authors:** Nattawadee Panyain, Aurélien Godinat, Aditya Raymond Thawani, Sofía Lachiondo-Ortega, Katie Mason, Sarah Elkhalifa, Lisa M. Smith, Jeanine A. Harrigan, Edward W. Tate

**Affiliations:** Department of Chemistry, Molecular Sciences Research Hub, Imperial College London London W12 0BZ UK nattawadee.panyain@epfl.ch e.tate@imperial.ac.uk; Mission Therapeutics Ltd, The Glenn Berge Building, Babraham Research Campus Babraham Cambridge CB22 3FH UK; The Francis Crick Institute London NW1 1AT UK

## Abstract

Ubiquitin carboxy-terminal hydrolase L1 (UCHL1), a deubiquitinating enzyme (DUB), is a potential drug target in various cancers, and liver and lung fibrosis. However, *bona fide* functions and substrates of UCHL1 remain poorly understood. Herein, we report the characterization of UCHL1 covalent inhibitor **MT16-001** based on a thiazole cyanopyrrolidine scaffold. In combination with chemical proteomics, a closely related activity-based probe (**MT16-205**) was used to generate a comprehensive quantitative profile for on- and off-targets at endogenous cellular abundance. Both compounds are selective for UCHL1 over other DUBs in intact cells but also engage a range of other targets with good selectivity over the wider proteome, including aldehyde dehydrogenases, redox-sensitive Parkinson's disease related protein PARK7, and glutamine amidotransferase. Taken together, these results underline the importance of robust profiling of activity-based probes as chemical tools and highlight the cyanopyrrolidine warhead as a versatile platform for liganding diverse classes of protein with reactive cysteine residues which can be used for further inhibitor screening, and as a starting point for inhibitor development.

## Abstract 2

We report the characterization of a UCHL1 covalent inhibitor based on a thiazole cyanopyrrolidine scaffold and a closely-related activity-based probe (ABP) which was used to generate a quantitative profile for on- and off-targets in human cells.

## Graphical abstract

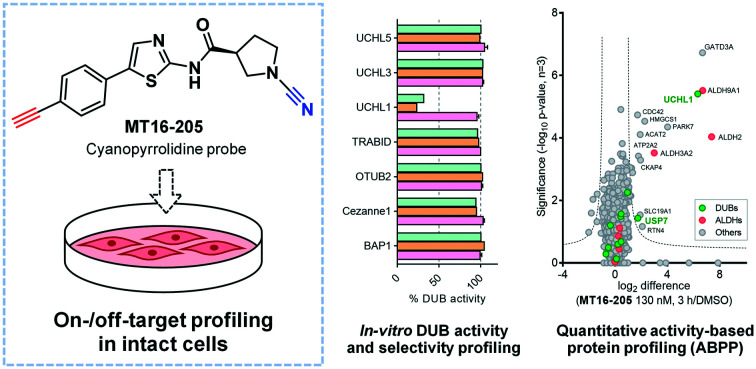

## Introduction

Protein ubiquitination, the covalent attachment of the 76-amino acid protein ubiquitin (Ub) to protein substrates predominantly *via* an isopeptide bond to lysine, is a global post-translational modification (PTM) in eukaryotic cells.^1^ The resulting linear or branched Ub chain conjugates regulate the timing and magnitude of protein degradation as a key component of the ubiquitin–proteasome system (UPS), alongside other important processes including protein trafficking, signalling and transcriptional regulation. Ubiquitination is a highly dynamic process in which the action of the Ub ligase cascade (driven by E1, E2 and E3 ligases) may be counteracted by deubiquitinating enzymes (DUBs), proteases responsible for removal of Ub on target proteins, processing mono-Ub precursors, and recycling Ub oligomers. The UPS has emerged as a promising therapeutic target in varied indications, and several DUBs are well-validated drug targets.^2,3^

Approximately 100 human DUBs have been identified and assigned to seven distinct families: ubiquitin carboxy-terminal hydrolases (UCHs), ubiquitin-specific proteases (USPs/UBPs), ovarian tumour proteases (OTUs), Machado–Josephin domain-containing proteases (MJDs/Josephins), motif interacting with ubiquitin-containing novel DUB family (MINDYs), Zinc finger-containing ubiquitin peptidase 1 (ZUP1/ZUFSP) and JAB1/MPN/Mov34 metalloenzyme (JAMM/MPN).^2,3^ Dysregulation and/or mutation of these enzymes have been implicated in a wide range of diseases including cancer, fibrosis, neurodegenerative and infectious diseases.^4–7^

Ub-conjugated activity-based probes (Ub-ABPs) with a variety of electrophilic warheads have been developed to profile DUB activity, through selective trapping of the active site cysteine across multiple DUB families.^8^ Ub-ABPs have been used for identification and characterization of novel DUBs, as well as for DUB inhibitor screening, but are primarily applied in cell lysates due to their inability to enter intact cells. In contrast, small-molecule, cell-permeable DUB ABPs complement the strengths and weaknesses of Ub-ABPs. Whilst DUB-selective small molecule probes are more challenging to develop and exhibit narrower DUB profiles, they offer powerful tools for profiling and imaging DUB activity in cells^9,10^ or whole organisms,^11^ accounting for the impact of physiological localization, biomolecular interactions and tissue context. They offer the further advantage of identifying off-targets for a given DUB inhibitor scaffold which would not be identified with the highly DUB-specific Ub-ABPs, therefore potentially better predicting unintended effects of future therapeutic agents.^12^

Ubiquitin carboxy-terminal hydrolase L1 (UCHL1, also known as PGP9.5) belongs to the UCH family of DUBs with a Cys-His-Asp catalytic triad and an unstructured crossover loop.^13^ The unstructured loop covers the active site and selectively restricts the size of ubiquitinated substrates. UCHL1 is highly expressed in brain or neuronal lysates, forming approximately 5% of total brain protein where it is important for neuronal function. However, it is also overexpressed in multiple disease-related conditions including fibrosis^14^ and pancreatic, colorectal and invasive breast cancers.^15,16^ However, the full scope of UCHL1 substrates has yet to be identified *in vivo*, and its molecular function remains poorly understood.

**Fig. 1:**
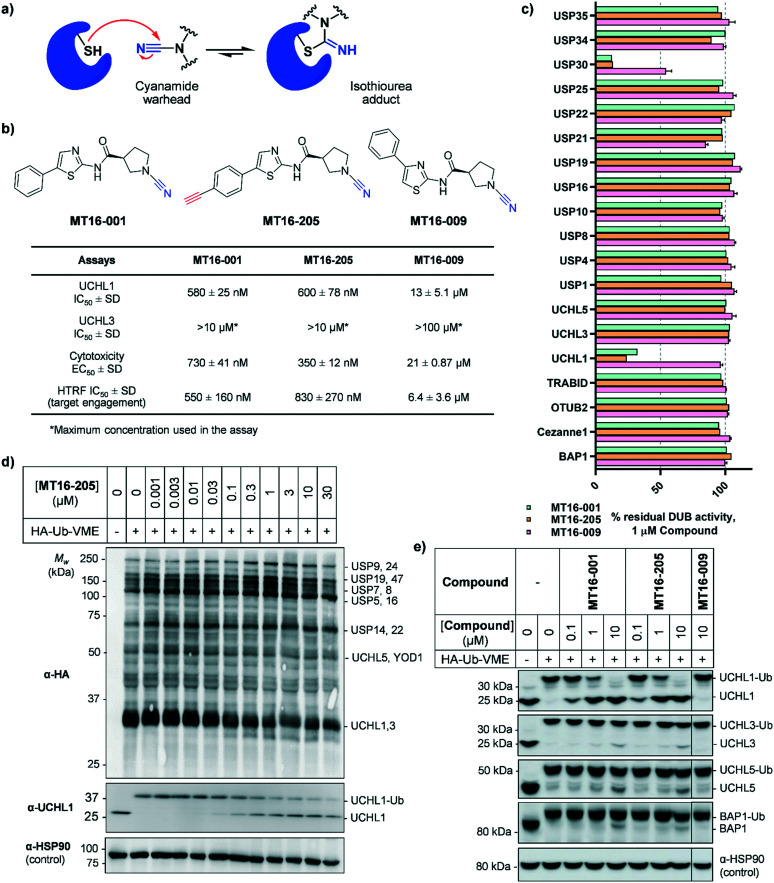
Biochemical and cellular characterization of compounds **MT16-001**, **MT16-205** and **MT16-009**. (a) Mechanism of cyanamide warhead labeling of a reactive cysteine residue. (b) Structures of compounds **MT16-001**, **MT16-205** and **MT16-009** and their activities in biochemical enzymatic, cellular cytotoxicity and target engagement assays. (c) Biochemical selectivity profiling of compounds **MT16-001**, **MT16-205** and **MT16-009** at 1 μM using fluorescence polarization (FP) and fluorescence intensity (FI) *in vitro* assays. (d and e) cellular target engagement of **MT16-205** using HA-Ub-VME ABP analyzed by immunoblotting against (d) HA antibody for whole DUB inhibition profiling or (e) DUB antibodies for selectivity profiling. HSP90 was used as a protein loading control.

Several UCHL1 inhibitors have been reported. Isatin *O*-acyl oxime LDN-57444 is most widely used as a putative tool to study the role of UCHL1 in both cellular and animal models,^17,18^ although recent reports have cast considerable doubt on the on-target activity of this compound.^10,11^ Several series of UCHL1 inhibitors have been reported in the patent literature, including cyanamide-containing compounds which likely react with the DUB active site cysteine residue forming an isothiourea adduct (Fig. 1a).^10,19,20^ These compounds represent an excellent foundation for ABPs selectively targeting UCHL1. For example, we recently reported a potent and selective cyanopyrrolidine-containing covalent inhibitor and an ABP for UCHL1 (IMP-1710), which selectively bind to Cys90 in the UCHL1 active site with minimal off-targets in intact cells.^10^ Whilst other reported cyanopyrrolidine UCHL1 ABPs may show good selectivity toward UCHL1 among DUBs they also exhibit suboptimal proteome selectivity, highlighting the importance of both on- and off-target profiling for ABPs.^11,21^

Herein, we report novel thiazole cyanopyrrolidine inhibitors targeting UCHL1, related to a previously reported UCHL1 ABP.^11^ In combination with chemical proteomics, an ABP based on this scaffold was used to generate a comprehensive quantitative profile for on- and off-targets of UCHL1 inhibitors at endogenous cellular abundance. We show that whilst this compound class is selective for UCHL1 over other DUBs in intact cells, it also engages a range of other targets with good selectivity over the wider proteome, including selected aldehyde dehydrogenases, redox-sensitive Parkinson's disease related protein DJ-1/PARK7, and glutamine amidotransferase. These data underline the importance of robust profiling of ABPs as chemical tools and highlight the cyanopyrrolidine warhead as a versatile platform for accessing diverse classes of protein with reactive cysteine residues.

## Results and discussion

**MT16-001** (example 1 in ref. 19), bearing a 5-phenylthiazole moiety linked to a cyanopyrrolidine warhead, and alkyne-containing analogue **MT16-205** (example 205 in ref. 19) were reported as UCHL1 inhibitors with IC_50_ < 1 μM (Fig. 1b) derived from a medicinal chemistry campaign.^19^ The alkyne present in **MT16-205** offers an ideal opportunity to undertake activity-based protein profiling (ABPP) in intact cells, facilitated by bioorthogonal copper(i)-catalyzed alkyne–azide cycloaddition (CuAAC) ligation to multifunctional capture reagents carrying fluorophores (*e.g.* TAMRA) and/or affinity tags (*e.g.* biotin).^22^ Regioisomer **MT16-009** (the (*S*)-enantiomer of racemic example 9 in ref. 19) bearing a 4-phenylthiazole is a less-potent analogue, with reported UCHL1 IC_50_ 10–30 μM, and was selected as a negative control for **MT16-001** due to its closely similar physicochemical properties.

We first examined UCHL1 and UCHL3 inhibitory potencies in a fluorescence polarization (FP) assay using a Ub-TAMRA substrate which is a mimic of the natural DUB substrate containing a native isopeptide bond of Ub-modified lysine.^23^ Compounds were pre-incubated with either recombinant UCHL1 or UCHL3 for 30 min, followed by addition of Ub-Lys-TAMRA substrate to initiate the reaction. Compounds **MT16-001** and **MT16-205** had similar UCHL1 IC_50_ of 580 ± 25 nM and 600 ± 78 nM respectively suggesting that the alkyne tag does not impact binding (Fig. 1b and S1†). Compound **MT16-009** was 20-fold less active enabling its use as a less active control. All three compounds failed to inhibit UCHL3 which shares 51% sequence identity with UCHL1, demonstrating impressive selectivity over the most closely related DUB enzyme. To profile DUB selectivity more broadly we screened each compound at 1 μM against 19 DUBs using FP and fluorescence intensity (FI) assays (Fig. 1c). Surprisingly, **MT16-001** and **MT16-205** inhibited both UCHL1 and the structurally unrelated DUB USP30 with similar potency, whilst negative control **MT16-009** was inactive at 1 μM against all of the tested DUBs except USP30 (50% inhibition), hinting at an interesting selectivity profile for this scaffold. The formation of a covalent adduct was explored by incubating recombinant UCHL1 (5 μM) with compounds **MT16-001** or **MT16-205** (13 μM) for 1 h at room temperature; the protein-compound mixture was analyzed by LC-ESI-MS revealing a single modification of UCHL1 for both **MT16-001** and **MT16-205** (Fig. S2†).

To identify working concentration ranges for the compounds in a cellular context, we determined the cytotoxicity of the compounds in human embryonic kidney cells (HEK293) which were subsequently used as a cell model for target identification in this study. **MT16-001** and **MT16-205** showed comparable EC_50_ of 730 ± 41 nM and 350 ± 12 nM at 72 h (Fig. S3†). The compounds affected cell proliferation in a steep dose–response, with negligible toxicity below 100 nM and maximum impact at *ca.* 2 μM, suggesting that alkyne-tagged **MT16-205** retains similar biological activity to the parent compound **MT16-001**. Interestingly, **MT16-009** had a similarly steep response, but with EC_50_ > 20 μM in HEK293 cells. Experiments with IMP-1710 have previously shown that on-target inhibition of UCHL1 is not strongly linked to cytotoxicity,^10^ and the steep response to the present compounds suggests the presence of off-targets which impart combinatorial toxicity at higher concentrations.

To assess cellular target engagement for **MT16-001**, **MT16-205** and **MT16-009**, a conventional Ub-based ABP (HA-Ub-VME) was applied with two in-lysate readouts, homogeneous time resolved fluorescence (HTRF) and immunoblotting.^24,25^ In the HTRF assay, human breast cancer cells (Cal51) stably expressing FLAG-UCHL1 were incubated with different concentrations of each compound for 1 h at 37 °C, and the cells washed, lysed, and incubated with HA-Ub-VME substrate for 30 min at room temperature. HTRF analysis of HA-Ub-VME labeling revealed that compounds **MT16-001** and **MT16-205** effectively engaged UCHL1 in cells, with similar IC_50_ of 550 ± 160 nM and 830 ± 270 nM, respectively (Fig. S4†), in line with biochemical UCHL1 IC_50_ (Fig. S1†), whilst as expected **MT16–009** was less potent at 6.4 ± 3.6 μM.

HA-Ub-VME was also applied to broader DUB profiling by immunoblot analysis, whereby HEK293 cells, which endogenously express UCHL1, were treated with varying concentrations of compound and lysed with a buffer which maintains DUB enzyme activity in lysates. Incubation with HA-Ub-VME to form a covalent adduct between probe and the reactive cysteine of DUBs was followed by separation of proteins on SDS-PAGE and immunoblotting using anti-HA antibody for pan-DUB profiling or selected DUB antibodies (such as UCHL1) for selective profiling taking advantage of the molecule weight increase on reaction with the ABP.^25^ Global protein labeling showed a modest decrease in intensity in response to increased concentrations of **MT16-205** specifically for the band between 25–37 kDa where HA-Ub-VME ABP-modified UCHL1 and UCHL3 enzymes migrate (Fig. 1d). The decrease in HA-Ub-VME labeling of UCHL1 was confirmed by anti-UCHL1 immunoblotting, comparing two bands of UCHL1 at 25 kDa and HA-Ub-VME-modified UCHL1 at 34 kDa. Increasing concentration of **MT16-205** decreased Ub probe-modified UCHL1, while unmodified UCHL1 band intensity increased in a concentration-dependent manner (Fig. 1d). This analysis suggested that **MT16-205** covalently bound at the active site of UCHL1, preventing Ub ABP adduct formation. Further immunoblots showed excellent selectivity of **MT16-001** and **MT16-205** for UCHL1 across the UCH family: UCHL1, UCHL3, UCHL5 and BAP1, whilst 10 μM **MT16-009** failed to engage any of these DUBs (Fig. 1e).

**Fig. 2:**
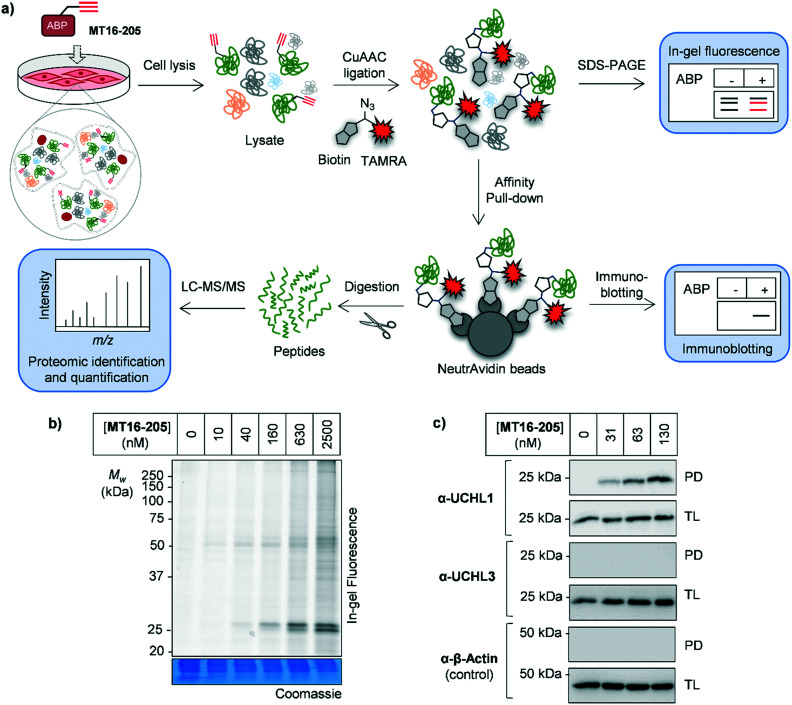
Activity-based protein profiling (ABPP) using **MT16-205** in HEK293 cells (a) activity-based protein profiling (ABPP) workflow in combination with mass-spectrometry proteomics. Briefly, cells are incubated with ABP and lysed. The ABP-labeled proteins are ligated with a capture reagent bearing a fluorophore (TAMRA) and/or affinity tag (biotin) *via* CuAAC. Labeled proteins are visualized by in-gel fluorescence analysis or affinity enriched and analyzed by immunoblotting. To identify and quantify the labeled proteins, enriched proteins are digested, resulting in peptides which can be analyzed by nanoLC-MS/MS. (b) In-gel fluorescence analysis of ABP **MT16-205** labeling. HEK293 cells were incubated with 10, 40, 160, 630 or 2500 nM **MT16-205** for 3 h. The lysate was ligated to capture reagent AzTB, separated by SDS-PAGE and visualized by in-gel fluorescence. (c) Affinity enrichment of ABP-labeled proteins was performed using streptavidin magnetic beads. The total lysate before-pulldown (TL) and pulldown (PD) samples were separated by SDS-PAGE and analyzed by in-gel fluorescence and immunoblotting using the indicated antibodies. β-Actin was used as a protein loading control.

As noted above, although Ub-based probes have been widely employed for DUB biology and inhibitor studies, they are membrane-impermeable and do not provide insight into targets outside the ‘DUBome’. We therefore turned to direct target engagement analysis using cell-permeable alkyne-tagged probe **MT16-205** in HEK293 cells by in-gel fluorescence and immunoblot. Cells were incubated with **MT16-205** at varying concentrations or vehicle control (DMSO) for indicated times, cells lysed and proteins subsequently ligated to capture reagent azido–TAMRA–biotin (AzTB, Fig. S5†)^26^ by CuAAC (Fig. 2a). TAMRA-labeled proteins were visualized by in-gel fluorescence scanning, and to confirm target engagement labeled proteins were affinity enriched on streptavidin beads and immunoblotted against the appropriate antibodies.

The isothiourea formed following cyanamide warhead attack at on active site cysteine has the potential to undergo subsequent hydrolysis or elimination,^10,27^ and we found that the inhibitor–enzyme complex was unstable under protein denaturing conditions including high temperature or strong reducing conditions (β-mercaptoethanol, BME) in protein sample loading buffer. This instability leads to a loss of fluorescence labeling and could be avoided by decreasing the concentration of BME in the sample loading buffer to 0.3% (v/v) final concentration and foregoing boiling (Fig. S6†). In-gel fluorescence analysis under these milder sample handling conditions shows UCHL1 labeling around 25 kDa within 3 h with detectible engagement at 40 nM **MT16-205** (Fig. 2b). Small molecule labeling selectivity was confirmed by biotin pulldown and immunoblotting against UCHL1 and UCHL3, with only UCHL1 significantly enriched by ABP **MT16-205** at concentrations as low as 31 nM (Fig. 2c). Whilst these data suggested that the band observed at 25 kDa in in-gel fluorescence analysis is probe-labeled UCHL1, higher concentrations of ABP **MT16-205** appear to bind a broader spectrum of other proteins in cells, implying the presence of significant off-targets.

To identify and quantify the protein targets of **MT16-205**, we implemented an unbiased quantitative chemical proteomic profiling workflow in HEK293 cells (Fig. 2a). We first performed a time-course experiment by incubating cells with 130 nM **MT16-205** or vehicle control (DMSO) for 3, 6 or 18 h, in order to follow covalent adduct formation between compound and protein targets over time. After probe treatment in intact cells, cells were lysed and proteins ligated to a trypsin-cleavable capture reagent azido-arginine-biotin, (AzRB, Fig. S5†)^26^ by CuAAC, and labeled proteins enriched on NeutrAvidin agarose beads, reduced and alkylated, and digested on-bead with trypsin. Tryptic peptides were then desalted prior to analysis by nanoscale liquid chromatography tandem mass spectrometry on a high-resolution QExactive orbitrap mass spectrometer (nanoLC-MS/MS).

**Fig. 3:**
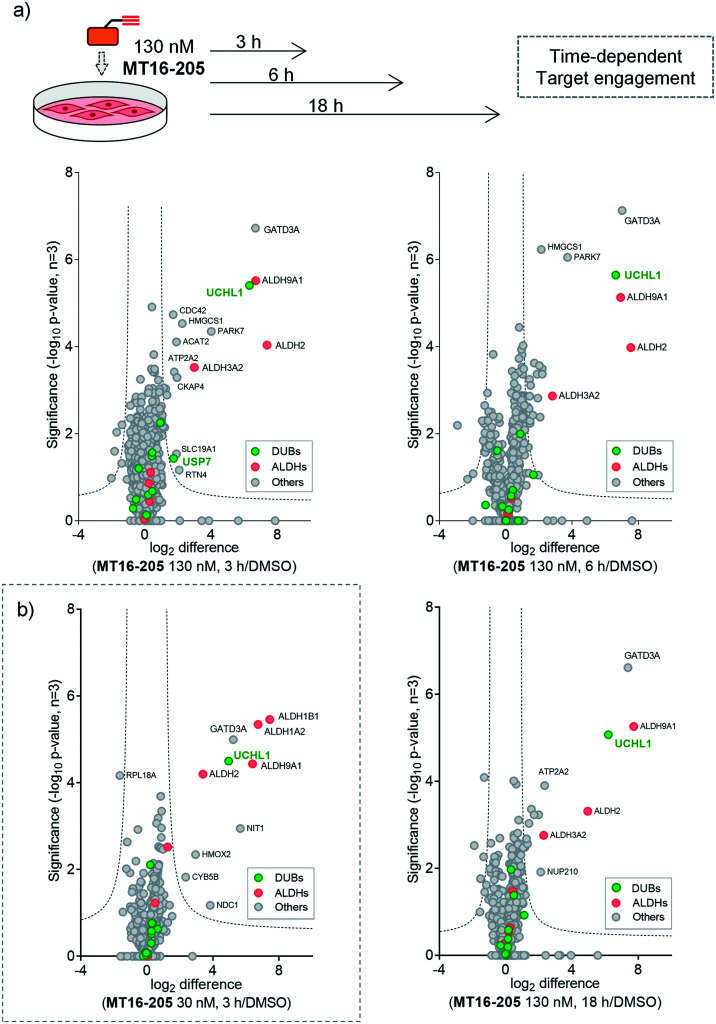
Target identification and quantification of ABP **MT16-205** labeling in HEK293 cells using LFQ-chemical proteomics (a) time- and (b) concentration-dependent ABPP experiments. Volcano plots show the fold change (log_2_ difference) and significance (−log_10_ *p*-value) between **MT16-205** and vehicle control (DMSO) at different incubation times (3, 6 or 18 h) and concentrations (30 or 130 nM), using a two-sample *t*-test (three biological replicates, permutation-based FDR = 0.01, *S*_0_ = 1). DUBs = green; ALDHs = red.

More than 1500 proteins were identified in the time-course experiment, but volcano plots for enriched protein profiling at different incubation times showed that only UCHL1 was enriched by **MT16-205** across the DUB family (Fig. 3a, ESI† S1); however, USP30 was not detected in this proteomics experiment. Engagement of UCHL1 was similar across all time points with saturated labeling at 3 h, consistent with in-gel fluorescence data (Fig. S7†). Comparison of statistically significantly enriched proteins at each time point (Table S1;† FDR of 1% and *S*_0_ of 1) showed that **MT16-205** targets specific proteins outside the DUB family with impressive selectivity over the >1500 proteins profiled. Strikingly, **MT16-205** engaged the aldehyde dehydrogenase (ALDH) family, particularly ALDH9A1, ALDH2 and ALDH3A2 at almost the same intensity as UCHL1 (Fig. 3a). ALDHs are a superfamily of enzymes that catalyze oxidation of aldehydes to carboxylic acids, and have been the subject of inhibitor development campaigns^28^ resulting in irreversible inhibitors for direct binding at the catalytic cysteine of the enzyme class; in-gel fluorescence labeling observed around 50–60 kDa matches to the size of ALDHs (Fig. 2b). Parkinson disease protein 7 (PARK7, also called protein deglycase DJ-1), which also carries a catalytic cysteine, was enriched by **MT16-205**, a result in line with recently reported key off-target of a related UCHL1 fluorescent ABP.^11^ The molecular weight of PARK7 correlates with in-gel fluorescence labeling around 20–25 kDa (Fig. 2b). A further interesting target was glutamine amidotransferase-like class 1 domain-containing protein 3A (GATD3A or C21orf33), a highly expressed enzyme in mitochondria, and the most significantly enriched protein across all conditions. However, the weak fluorescence observed around 25–37 kDa by in-gel fluorescence analysis suggests that labeling of some **MT16-205** targets may be particularly unstable, highlighting a potential advantage of mild on-bead proteomic analysis for target identification.

We next identified the protein targets at a lower concentration to minimize off-targets of the ABP by incubating 30 nM of **MT16-205** in HEK293 cells for 3 h, following the same chemical proteomic workflow (Fig. 2a). The enriched proteins found in this experiment were similar to the prior experiment; however, fewer off-targets were observed at 30 nM of **MT16-205** (Fig. 3b), compared to 130 nM, with UCHL1, ALDHs and GATD3A significantly and selectively enriched by **MT16-205** with >16-fold enrichment compared to vehicle control at 30 nM and >64-fold at 130 nM, suggesting that these targets are enriched in a concentration-dependent manner.

**Fig. 4:**
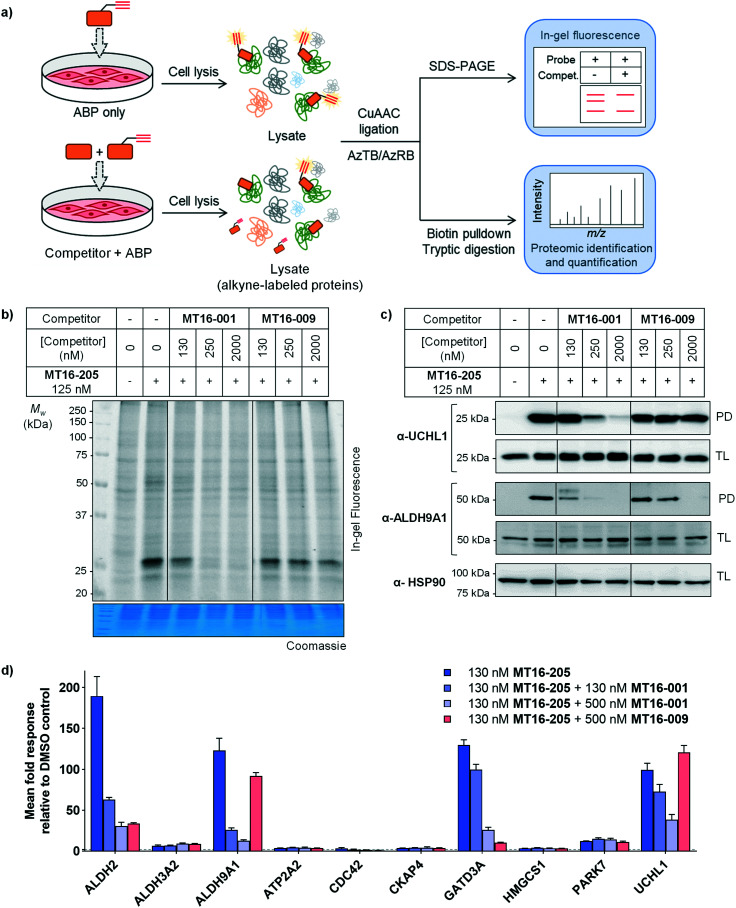
In-gel fluorescence, immunoblot and proteomics analysis by competitive ABPP in HEK293 cells (a) general workflow for competitive ABPP. (b) In-gel fluorescence analysis of competitive ABPP of **MT16-001** and **MT16-009** against ABP **MT16-205** in HEK293 cells. Protein loading was assessed by Coomassie blue staining (c) the ABP-labeled proteins were enriched using streptavidin magnetic beads. The total lysate before pulldown (TL) and pulldown (PD) samples were analyzed by immunoblotting using the indicated antibodies. HSP90 was used as a protein loading control. (d) The labeled proteins were analyzed by nanoLC-MS/MS. The data was processed in MaxQuant and analyzed in Perseus. Data represent mean ± SEM (three biological replicates). The hits were selected in the ABP-enriched proteins with the fold-change >4 and *p*-values >1. The selected hits were normalized to vehicle controls.

Competitive activity-based protein profiling (ABPP) was performed to identify the protein targets of the parent compound or UCHL1 inhibitor, and to remove non-specific binders resulting from protein–protein interactions in the biotin pulldown step (Fig. 4a). HEK293 cells were pre-incubated with varying concentrations of parent compound (**MT16-001**), negative control (**MT16-009**) or vehicle control (DMSO) for 1 h, followed by addition of 130 nM ABP **MT16-205** for 3 h. After treatment, the lysate was processed following the ABPP workflow (Fig. 2a). The fluorescence labeling of ABP **MT16-205** was competed in a concentration-dependent manner by the parent compound **MT16-001** and marginally reduced by the negative control **MT16-009** only at the highest concentration (2 μM) (Fig. 4b). We then performed affinity enrichment and immunoblotting to confirm competition for UCHL1 labeling. The UCHL1 band was depleted under **MT16-001** treatment in a concentration-dependent manner and also ALDH9A1, which was one of the off-targets of **MT16-205** in HEK293 cells (Fig. 4c). The ALDH9A1 band was nearly entirely out-competed by **MT16-001** at 250 nM. Interestingly, 2 μM **MT16-009** could compete probe binding to ALDH9A1 but not to UCHL1, which suggests that the binding site of ALDH9A1 has a higher tolerance to substitutions on the thiazole ring.

To identify and quantify inhibitor targets across the human proteome, competitive ABPP was performed using label-free quantitative (LFQ) proteome analysis. Over 1500 proteins were identified; 24 proteins were significantly enriched by **MT16-205** (FDR of 1% and *S*_0_ of 1). The top 10 targets of **MT16-205** with fold-change >4 were selected to explore the response to **MT16-001** and **MT16-009** treatment (Fig. 4d, ESI† S2). Four targets, ALDH2, ALDH9A1, GATD3A and UCHL1, responded to parent compound **MT16-001** in a concentration dependent manner. However, ALDH2 and GATD3A demonstrated response to the negative control **MT16-009**, suggesting that both proteins are common targets of this scaffold. ALDH9A1 was slightly inhibited by **MT16-009** at 500 nM while the immunoblot analysis of ALDH9A1 showed this protein was outcompeted at 2 μM (Fig. 4c). Consistent with previous gel-based assays, no response was observed for UCHL1 in treatment with negative control **MT16-009**.

Covalent modification of the multiple targets of **MT16-001** may lead to downstream phenotypic effects, and to build on insights from the proteomic analysis the impact of parent compound **MT16-001** was therefore examined at the whole proteome level. HEK293 cells were treated with increasing concentrations of **MT16-001** (2, 20, 200 or 2000 nM) for 3 h, and proteins from cell lysates processed and analyzed by nanoLC-MS/MS using 10-plex tandem mass tag (TMT) quantification. This proteomic analysis showed no effect at the whole proteome level from parent compound **MT16-001** treatment up to 2000 nM (Fig. S8, ESI† S3), suggesting that **MT16-001** causes limited acute disruption of downstream pathways.

## Conclusions

In this work, we characterized a potent small molecule UCHL1 inhibitor and ABP using biochemical enzymatic, cellular and proteomic analyses. Parent compound **MT16-001** and ABP **MT16-205** displayed high biochemical potency against UCHL1 and USP30, with nanomolar IC_50_ values. Cellular target engagement by HTRF and gel-based Ub-VME ABP assays further confirmed cell-permeability and selectivity of these compounds leading to further cellular validation experiments. Interestingly, proteomic profiling revealed that both UCHL1 and the ALDH family were targeted by **MT16-205**. Combining both gel-based and MS based-proteomic results, ALDH2, ALDH9A1, and GATD3A were confirmed as the genuine targets of **MT16-001**, **MT16-205** and **MT16-009** which suggests that these proteins can accommodate a similar inhibitor scaffold, whilst UCHL1 was shown to be a specific target of parent compound **MT16-001** and ABP **MT16-205**, but not negative control **MT16-009**. This suggests that the active site of UCHL1 accommodates only one regioisomer of the parent compound and changing the position of phenyl substituent causes a loss of activity for UCHL1, suggesting a vector for future exploration and optimization of UCHL1 inhibitors. Moreover, **MT16-001** presented reasonable phenotypic selectivity within the window over which selectivity was analyzed, with up to 2000 nM and 3 h incubation showing no effect on the overall proteome.

Competitive ABPP highlights the potential of identification and quantification to profile the on- and off-targets of the compounds in intact cells; whilst more than 10 proteins were enriched by ABP **MT16-205** at 130 nM (fold change >4 and *p*-values >1), only 4 were identified as genuine targets of the parent compound, **MT16-001**. The other targets may be off-targets of the ABP design or non-specific binding proteins from pulldown, which can occur as a background in this workflow; a competitive ABPP approach is useful in target ID campaigns as it can help to filter out such false positive targets and provide a high-confidence target list for the parent compound, offering a powerful tool for in-cell inhibitor profiling.

Our data suggest that compounds **MT16-001** and **MT16-205** are promising tools to study UCHL1, and that ABP **MT16-205** constitutes a useful probe with additional potential for profiling multiple proteins in the ALDH family. Whilst these compounds are neither as selective nor as potent as our recently disclosed UCHL1 inhibitor and ABP **IMP-1710**,^10^ they provide an interesting insight into ABP design for UCHL1 using a novel scaffold and demonstrate considerable scope for tuning selectivity across diverse protein families through variations on the cyanopyrrolidine warhead. Future optimization of **MT16-205** may ultimately provide a new class of potent UCHL1 inhibitors.

## Author contributions

N. P. undertook experiments, analyzed data and wrote the original draft of the manuscript. A. G. and A. R. T. contributed to synthesis of compounds. S. L. O. contributed to enzyme expression and assay data. K. M. performed biochemical screening assays, S. E. performed cellular activity probe assays, L. M. S. analyzed data and J. A. H supervised experiments. E. W. T. conceived and supervised the study. All authors contributed to the final version of the manuscript.

## Conflicts of interest

The authors declare the following competing financial interest(s): K. M., S. E., L. M. S., and J. A. H. are current or previous employees of Mission Therapeutics Ltd; E. W. T. is a Director and shareholder in Myricx Pharma Ltd.

## Supplementary Material

MD-012-D1MD00218J-s001

MD-012-D1MD00218J-s002

MD-012-D1MD00218J-s003

MD-012-D1MD00218J-s004
