# Supplementary material for: Activity-based protein profiling reveals deubiquitinase and aldehyde dehydrogenase targets of a cyanopyrrolidine probe
Source: RSC Med Chem. 2021 Aug 16;12(11):1935–43. doi: 10.1039/d1md00218j (PMC8597422; doi:10.1039/d1md00218j)

Electronic Supplementary Material (ESI) for RSC Medicinal Chemistry.
This journal is © The Royal Society of Chemistry 2021

Supplementary Information

Activity-based protein profiling reveals deubiquitinase and aldehyde dehydrogenase targets of a cyanopyrrolidine probe

Nattawadee Panyain,*[a] Aurélien Godinat,[a] Aditya Raymond Thawani,[a] Sofía Lachiondo-Ortega,[a] Katie Mason,[b] Sarah Elkhalifa,[b] Lisa M. Smith,[b] Jeanine A. Harrigan,[b] and Edward W. Tate*[ac]

[a] Department of Chemistry, Molecular Sciences Research Hub, Imperial College London, London, W12 0BZ, U.K.
[b] Mission Therapeutics Ltd, The Glenn Berge Building, Building 940, Babraham Research Campus, Babraham, Cambridge, CB22 3FH, U.K.
[c] The Francis Crick Institute, London, NW1 1AT, U.K.

* Corresponding author: e.tate@imperial.ac.uk (E.W. Tate)
nattawadee.panyain@epfl.ch (N. Panyain)

Table of contents

Supplementary Results S3
Supplementary Figures S4
Supplementary Tables...S15
Materials and Methods S18
Biological and Biochemical Methods and Proteomics S18
Chemical Method..S23
References S29
^{1}H and ^{13}C NMR Spectra S30

Supplementary Results

Fig. S1. Biochemical enzymatic assay of compounds..S4
Fig. S2. LC-ESI analysis of covalent adduct of MT16-001 or MT16-205 and UCHL1..........S5
Fig. S3. Cytotoxicity of compounds after 72 h treatment in HEK293...................................S6
Fig. S4. Cellular UCHL1 activity profiling of compounds using Homogeneous time resolved fluorescence (HTRF) assay..S7
Fig. S5. Structures of capture reagents; AzTB and AzRB...S8
Fig. S6. UCHL1-ABP MT16-205 adduct stability study S9
Fig. S7. In-gel fluorescence analysis of ABP MT16-205 labeling......................................S10
Fig. S8. Whole proteome analysis of parent compound MT16-001 treatment S11
Fig. S9 Uncropped gels/blots S12

Table S1. List of enriched proteins by LC-MS/MS analysis of ABP MT16-205 labeling......S16
Table S2. Primary and secondary antibodies used. S17

Supplementary Data Files (Separated Excel files)

Supplementary Data S1 Protein lists of ABP MT16-205 target engagement
Supplementary Data S2 Protein lists of competitive ABPP
Supplementary Data S3 Protein lists of whole proteome analysis

Supplementary Figures

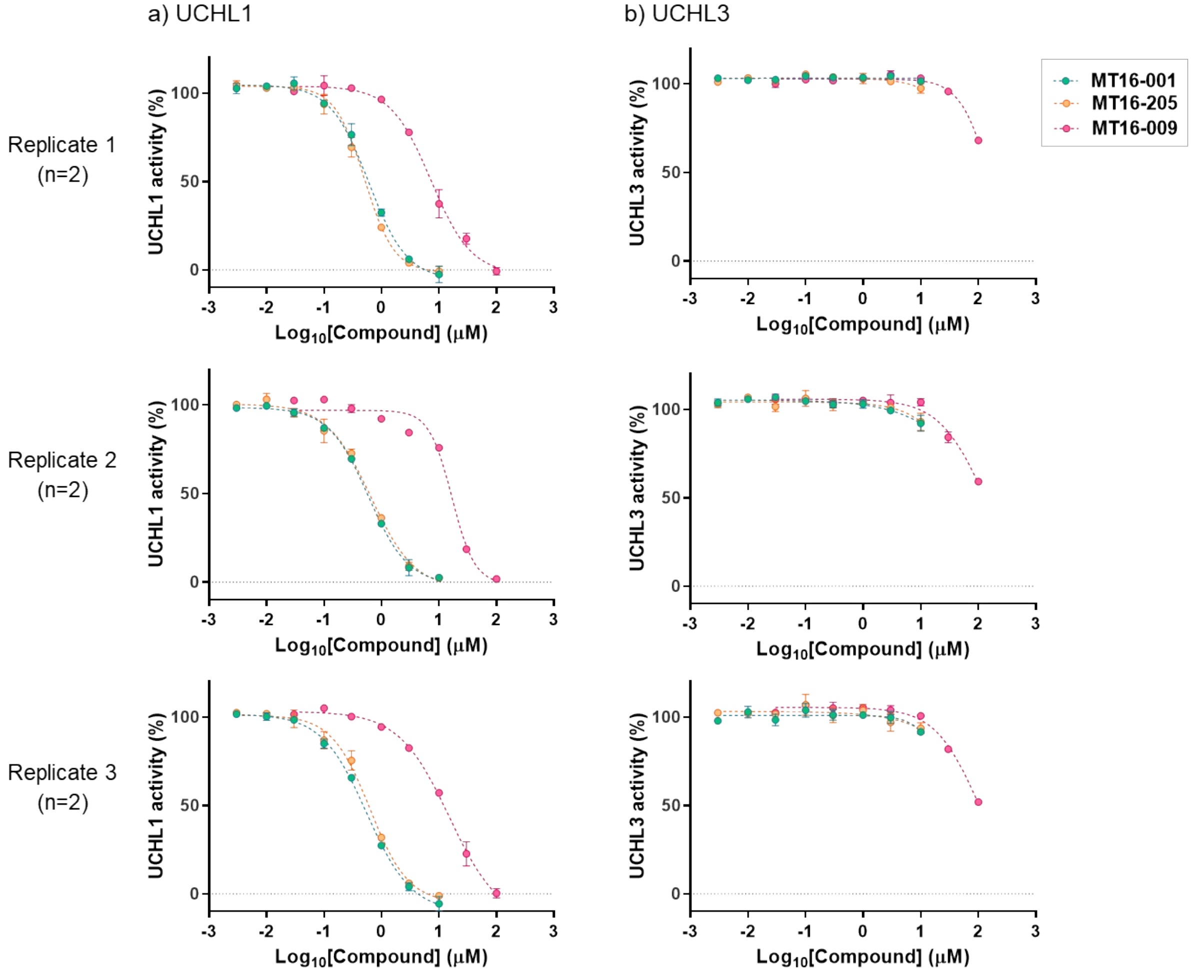

Compound	FP IC_{50} UCHL1			
	Replicate 1 (n=2)	Replicate 2 (n=2)	Replicate 3 (n=2)	Mean IC_{50} ± SD
**MT16-001**	600 nM	590 nM	550 nM	**580 ± 25 nM**
**MT16-205**	510 nM	650 nM	630 nM	**600 ± 78 nM**
**MT16-009**	7.2 μM	17 μM	15 μM	**13 ± 5.1 μM**

Compound	FP IC_{50} UCHL3			
	Replicate 1 (n=2)	Replicate 2 (n=2)	Replicate 3 (n=2)	Mean IC_{50} ± SD
**MT16-001**	>10 μM*	>10 μM*	>10 μM*	>10 μM*
**MT16-205**	>10 μM*	>10 μM*	>10 μM*	>10 μM*
**MT16-009**	>100 μM*	>100 μM*	>100 μM*	>100 μM*

*Maximum concentration used in the assay

Fig. S1. Biochemical enzymatic assay of compounds
(a) UCHL1 and (b) UCHL3 activities were measured using fluorescence polarization (FP) with Ub-Lys-TAMRA in triplicate. Recombinant UCHL1 or UCHL3 (5 nM) were incubated with 8-point dilutions of test compounds for 30 min, and measurement initiated by adding Ub-Lys-TAMRA (50 nM). IC_{50} was calculated from a four-parameter dose-response curve and represented as mean ± SD (three biological replicates).

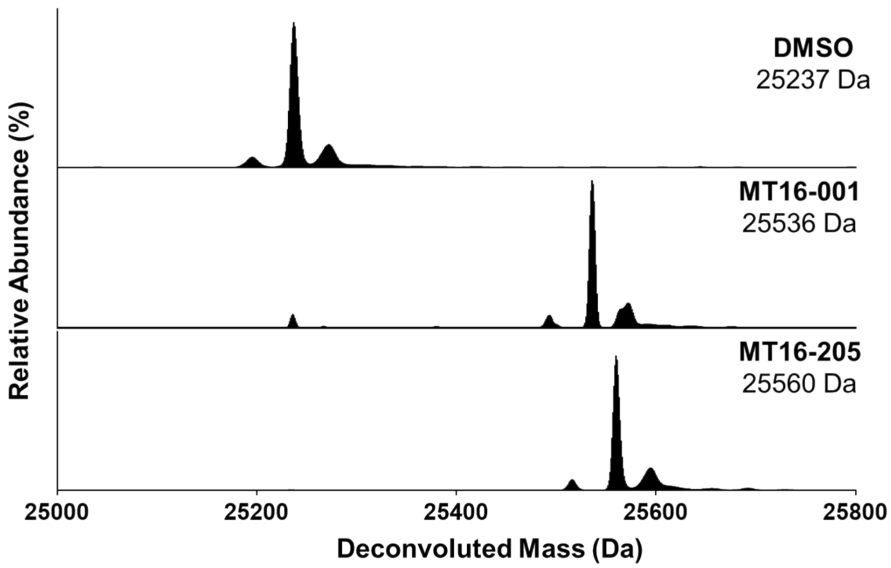

Fig. S2. LC-ESI analysis of covalent adduct of MT16-001 or MT16-205 and UCHL1
Recombinant UCHL1 (5 μM) was incubated with **MT16-001** or **MT16-205** (13 μM) for 1 h at room temperature. The protein-compound mixture was desalted and analyzed by LC-ESI-MS. The LC-ESI analysis demonstrates a single modification of UCHL1 for both **MT16-001** (expected mass $[M+H]^+$ 25536 Da, found 25536 Da) and **MT16-205** (expected mass $[M+H]^+$ 25560 Da, found 25560 Da).

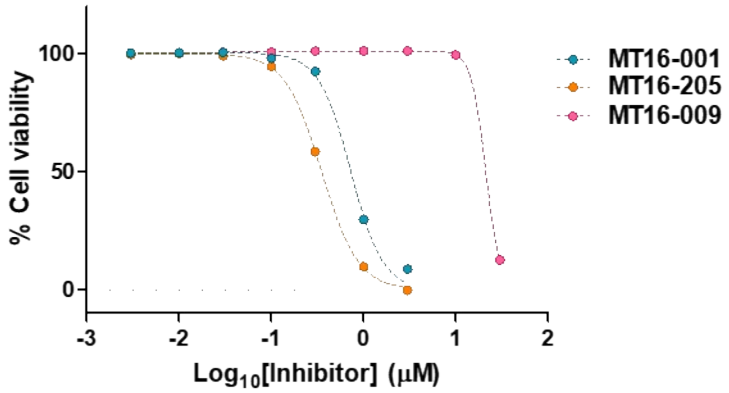

Compound	**EC$_{50}$** ± SD
**MT16-001**	730 ± 41 nM
**MT16-205**	350 ± 12 nM
**MT16-009**	21 ± 0.87 μM

Fig. S3. Cytotoxicity of compounds after 72 h treatment in HEK293
HEK293 cells measured by using IncuCyte live-cell image. HEK293 cells were seeded overnight at 37 ºC prior to incubate with compounds. Cell viability was measured by comparing the occupied area of cell images captured every 2 h for 72 h using an IncuCyte S3 live-cell time-lapse imaging device. The cell imaging was analyzed by IncuCyte analysis software module. Graphs represent mean ± SD (12 technical replicates). IC$_{50}$ were calculated from a four-parameter dose-response curve.

Replicate 1
(n=2)

Replicate 2
(n=2)

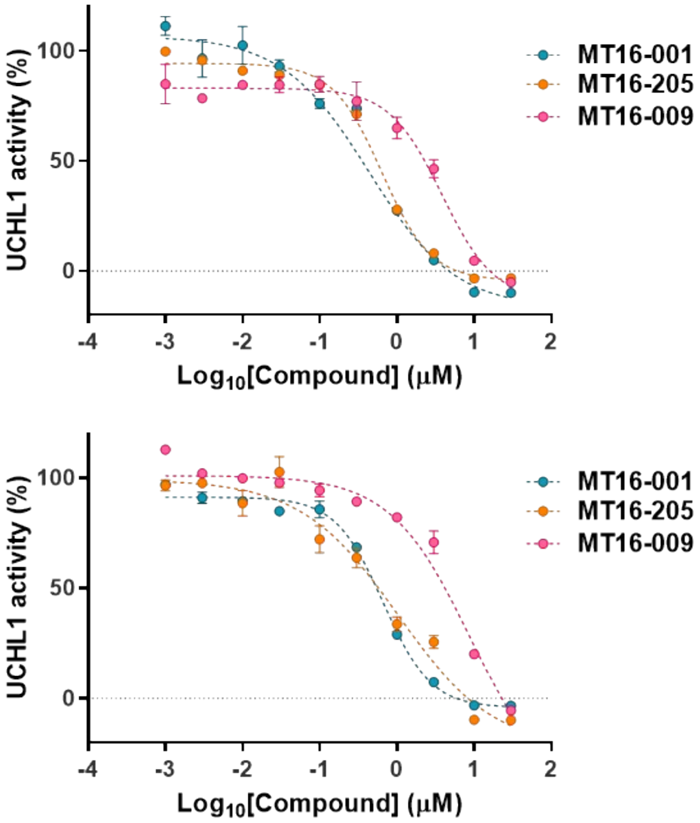

Compound	HTRF IC50		
	Replicate 1 (n=2)	Replicate 2 (n=2)	Mean IC_{50} ± SD
**MT16-001**	440 nM	660 nM	**550 ± 160 nM**
**MT16-205**	640 nM	1000 nM	**830 ± 270 nM**
**MT16-009**	3.8 μM	8.9 μM	**6.4 ± 3.6 μM**

Fig. S4. Cellular UCHL1 activity profiling of compounds using Homogeneous time resolved fluorescence (HTRF) assay
FLAG-UCHL1 was overexpressed in Cal51 cells and treated with compounds **MT16-001, MT16-205** or **MT16-009** for 1 h at 37ºC before lysis. Lysates were combined with HA-Ub-VME (100 nM) and incubated for 30 min at room temperature. Detection antibodies were added to the reaction plate and incubated overnight at 4ºC before measurement. HTRF ratios were calculated as described in Materials and Methods. IC_{50} was calculated from a four-parameter dose-response curve and represented as mean ± SD (two biological triplicates).

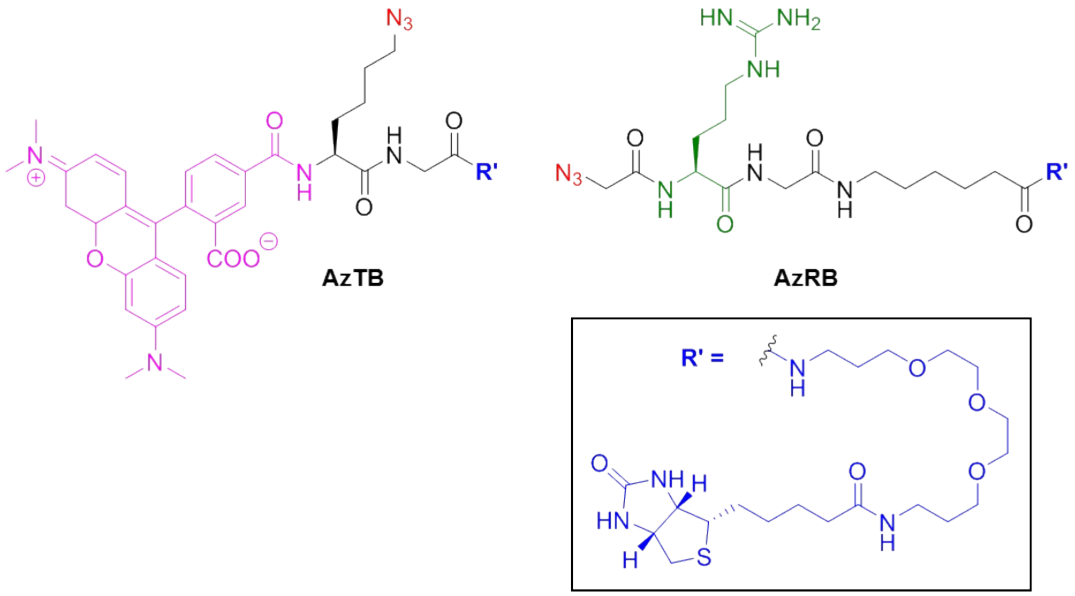

Fig. S5. Structures of capture reagents; AzTB and AzRB

Structures of azido-TAMRA-biotin (AzTB) and azido-arginine-biotin (AzRB). Capture reagents contain key functional moieties; an azide (red) for copper(I)-catalyzed alkyne-azide cycloaddition (CuAAC) reaction, a TAMRA fluorophore (pink) for in-gel fluorescence visualization, a biotin affinity handle (blue) for enrichment and purification, and an arginine residue (green) for on-bead trypsin digestion.

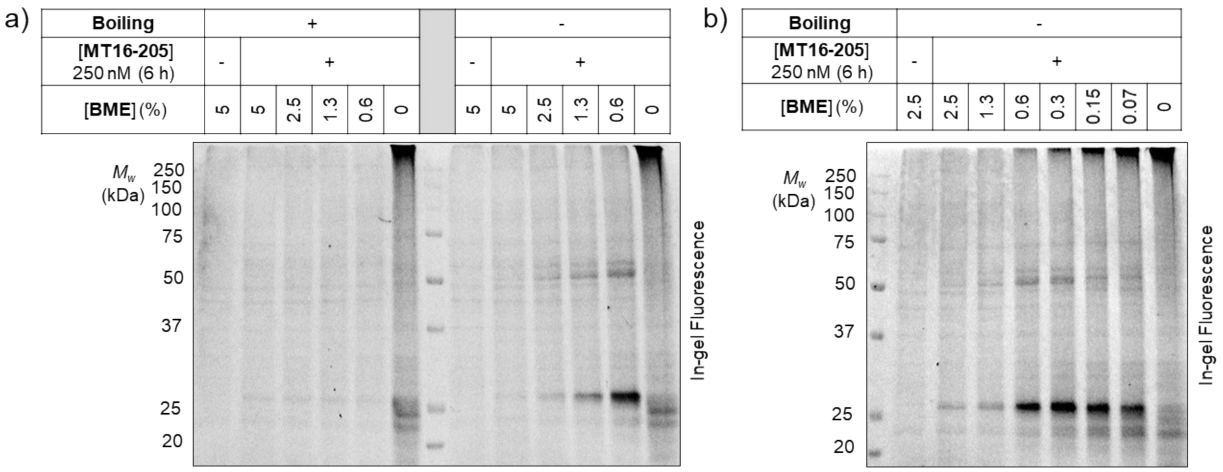

Fig. S6. UCHL1-ABP MT16-205 adduct stability study

The TAMRA-labeled proteins from HEK293 cells treated with 250 nM of **MT16-205** (6 h) were visualized by in-gel fluorescence under different denaturing conditions. (a) Comparison of fluorescence labeling under with and without boiling conditions in the presence of various concentrations of β-mercaptoethanol (BME). (b) Optimization of BME concentration measured by fluorescent intensity.

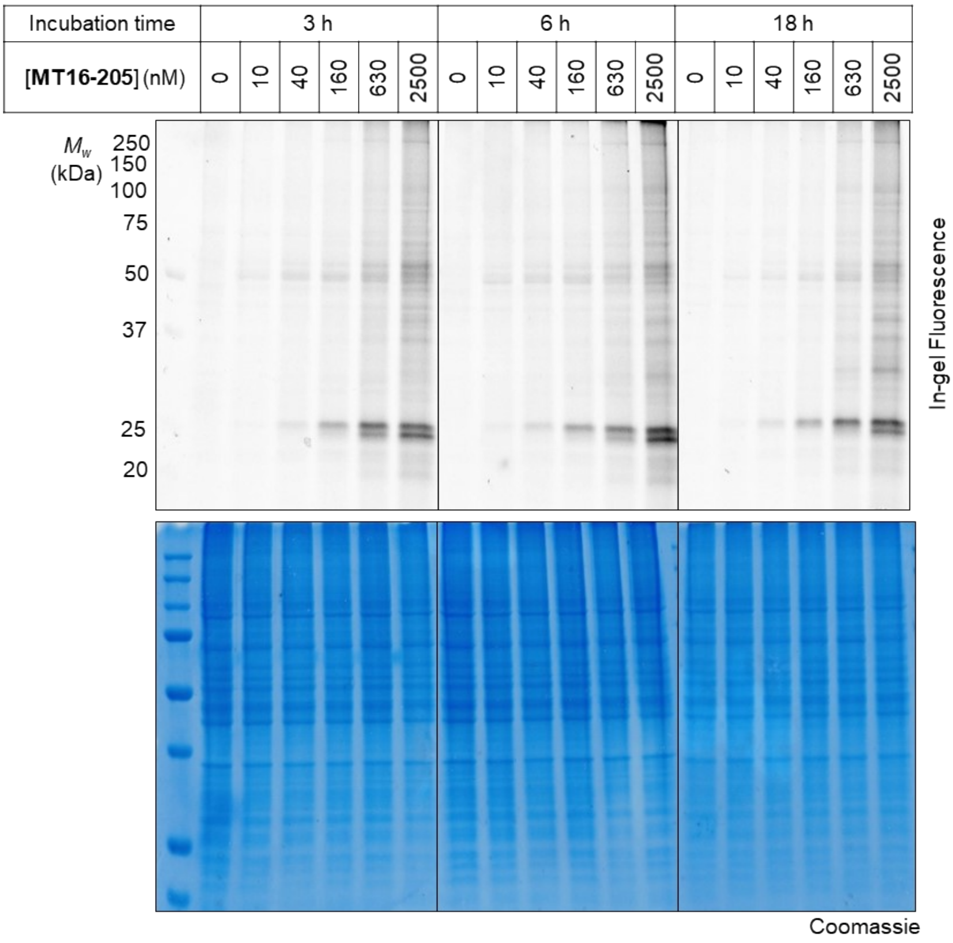

Fig. S7. In-gel fluorescence analysis of ABP MT16-205 labeling
HEK293 cells were incubated with 10, 40, 160, 630 or 2500 nM of **MT16-205** for 3, 6 or 18 h. The lysate was ligated to capture reagent AzTB, separated by SDS-PAGE and visualized by in-gel fluorescence.

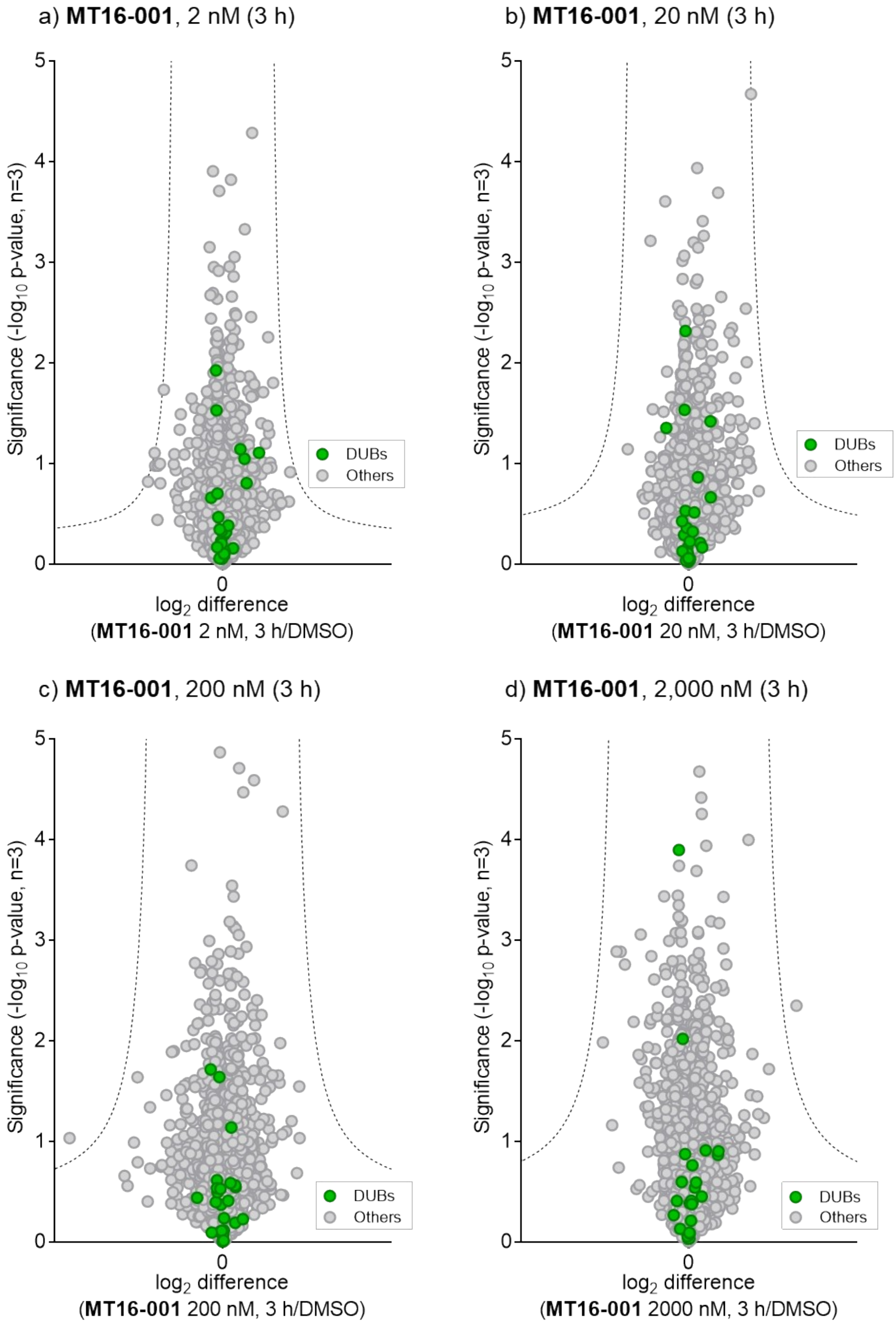

Fig. S8. Whole proteome analysis of parent compound MT16-001 treatment
HEK293 cells were treated with **MT16-001** (2, 20, 200 or 2000 nM) or vehicle (DMSO) for 3 h. Following cell lysis, reduction and alkylation, the samples were tryptic digested and labeled with 10-plex tandem mass tag (TMT) reagents, fractionated and analyzed by LC-MS/MS. Data was processed in MaxQuant and analyzed in Perseus as described in Materials and Methods. Volcano plots showing the fold change ($\log_2$ difference) and significance ($-\log_{10}$ p-value) between protein response at different concentration of **MT16-001** (2, 20, 200 or 2000 nM) and DMSO control using a two-sample t-test (biological replicates, Permutation-based FDR = 0.01, S_0 = 1). **MT16-001** treatment does not affect protein abundance across the whole proteome.

Fig. S9 Uncropped gels/blots

- Uncropped gels/blots for Fig. 1

Fig. 1d Immunoblot

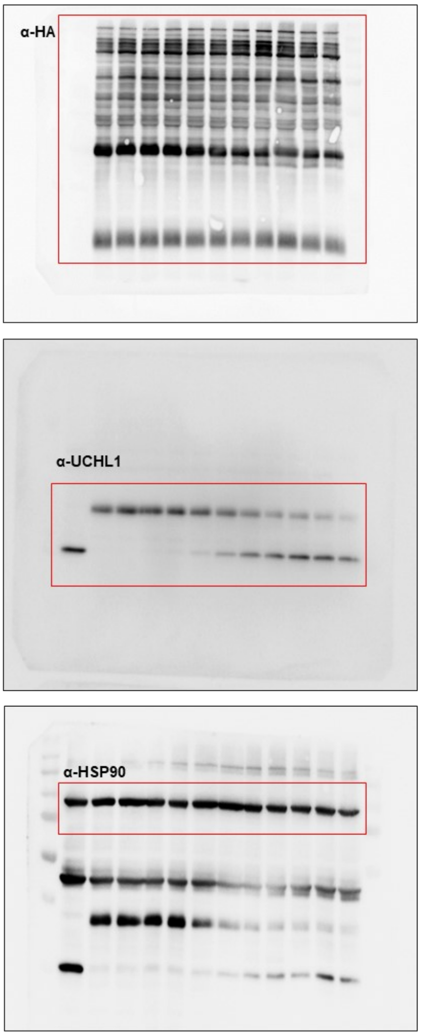

Fig. 1e Immunoblot

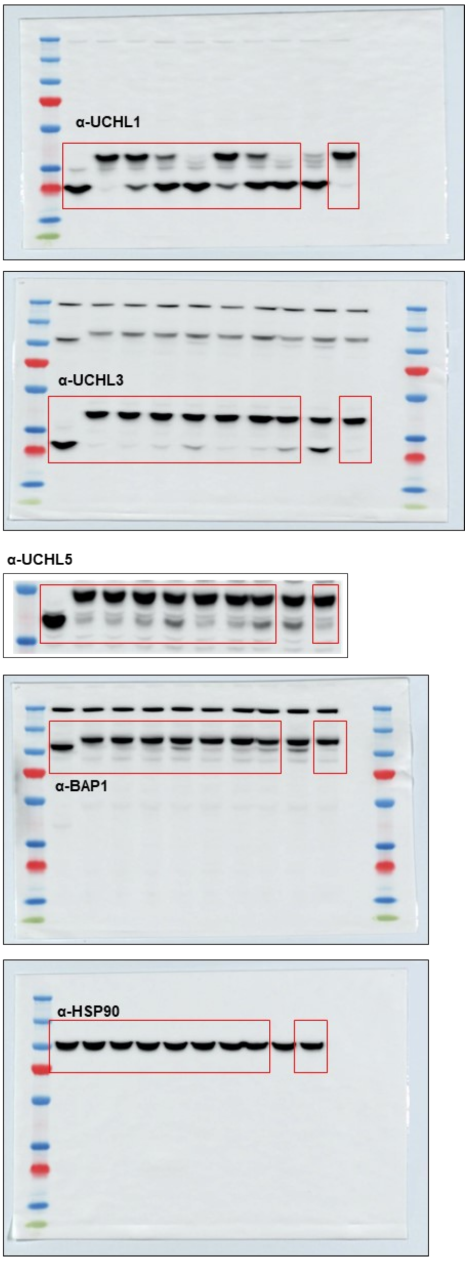

- Uncropped gels/blots for Fig. 2

Fig. 2d_In-gel Fluorescence

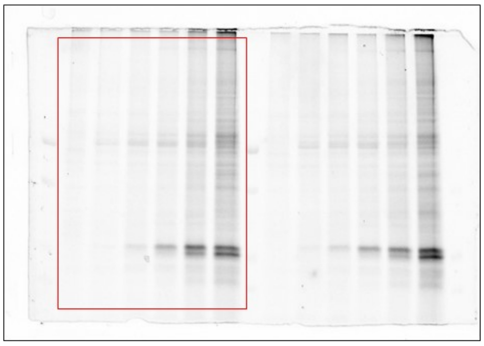

Coomassie

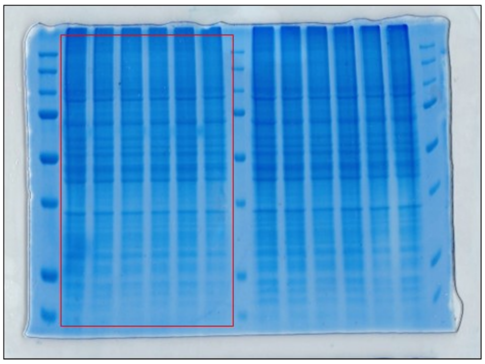

Fig. 2c immunoblot

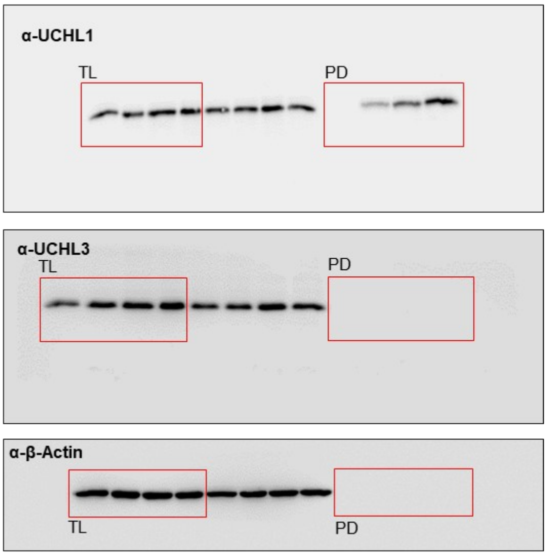

- Uncropped gels/blots for Fig. 4

Fig. 4b_In-gel Fluorescence

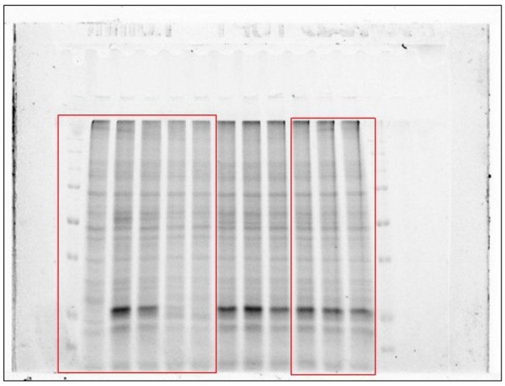

Coomassie

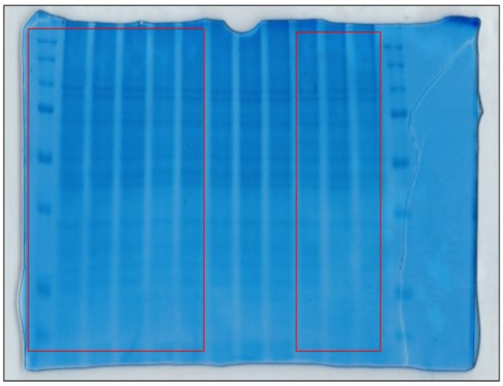

Fig. 4c immunoblot

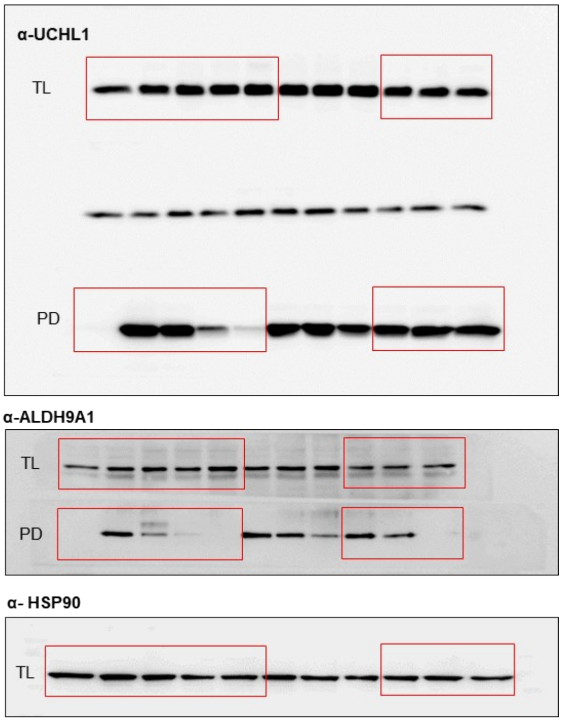

- Uncropped gels/blots for Fig. S6

In-gel Fluorescence

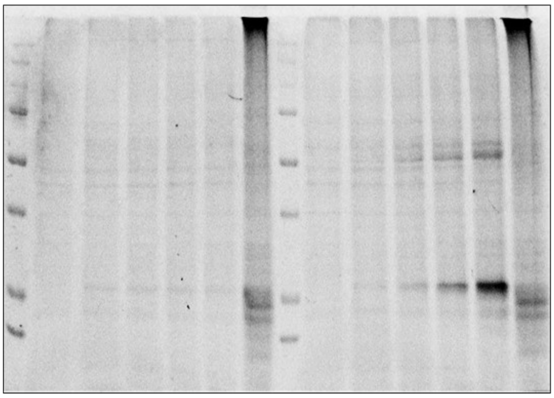

In-gel Fluorescence

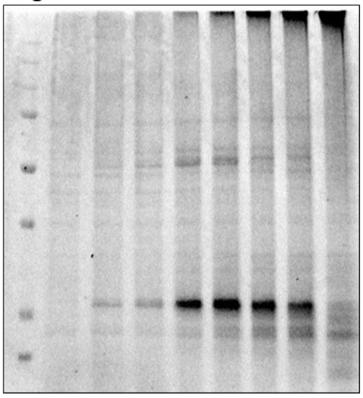

- Uncropped gels/blots for Fig. S7

In-gel Fluorescence

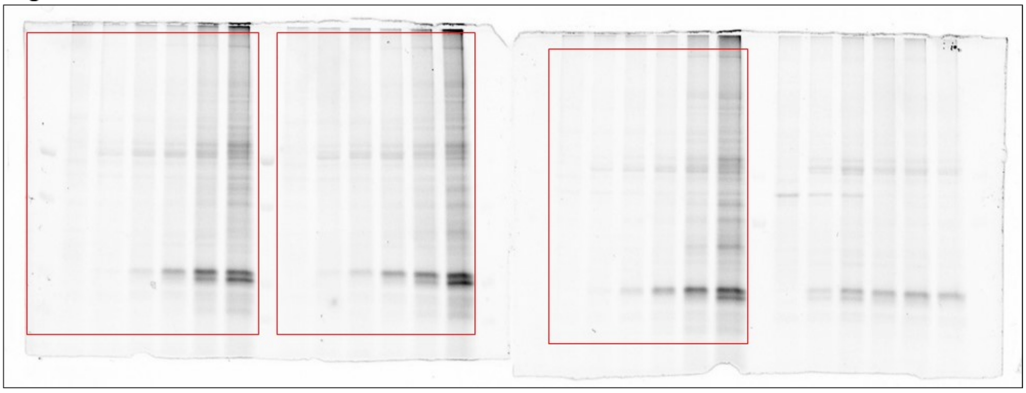

Coomassi

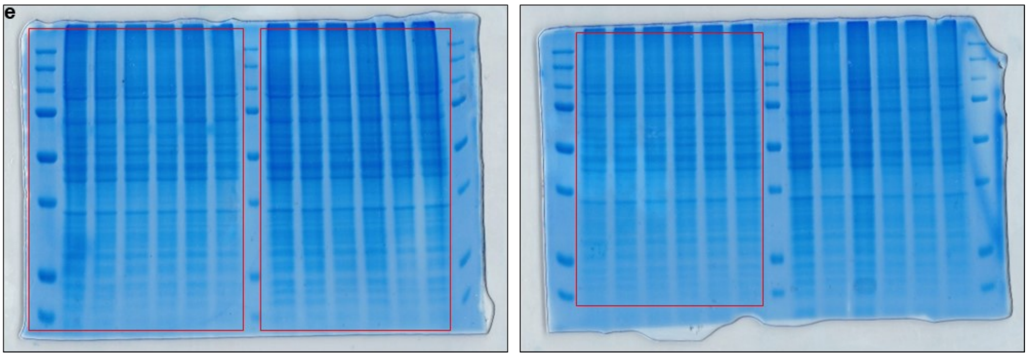

Supplementary Tables

Gene name	Protein name	Significant (FDR 0.01, S_0: 1)			Mass (kDa)
		3 h	6 h	18 h	
ACAT2	Acetyl-CoA acetyltransferase, cytosolic	+	+		60
ALDH2	Aldehyde dehydrogenase, mitochondrial	+	+	+	56
ALDH3A2	Fatty aldehyde dehydrogenase	+	+	+	55
ALDH9A1	4-trimethylaminobutyraldehyde dehydrogenase	+	+	+	54
ATP2A2	Sarcoplasmic/endoplasmic reticulum calcium ATPase 2	+	+	+	115
CDC42	Cell division control protein 42 homolog	+	+		21
CFL1	Cofilin-1		+		19
CKAP4	Cytoskeleton-associated protein 4	+	+	+	66
DDX39B	Spliceosome RNA helicase DDX39B		+		49
HACD3	Very-long-chain (3R)-3-hydroxyacyl-CoA dehydratase 3		+	+	43
HMGCS1	Hydroxymethylglutaryl-CoA synthase, cytoplasmic	+	+		57
NUP210	Nuclear pore membrane glycoprotein 210			+	205
PARK7	Protein deglycase DJ-1	+	+	+	20
PDIA6	Protein disulfide-isomerase A6		+		48
PNP	Purine nucleoside phosphorylase		+		32
PREP	Prolyl endopeptidase			+	81
RTN4	Reticulon-4	+	+		130
TFRC	Transferrin receptor protein 1		+		85
TIMM50	Mitochondrial import inner membrane translocase subunit TIM50		+		40
TOMM22	Mitochondrial import receptor subunit TOM22 homolog		+		16
TOMM40	Mitochondrial import receptor subunit TOM40 homolog	+	+	+	38
TXNDC5	Thioredoxin domain-containing protein 5		+		48
UCHL1	Ubiquitin carboxyl-terminal hydrolase L1	+	+	+	25
USP7	Ubiquitin carboxyl-terminal hydrolase 7	+			128
VDAC2	Voltage-dependent anion-selective channel protein 2	+	+	+	32

VDAC3	Voltage-dependent anion-selective channel protein 3	+	+	+	29
GATD3A	Glutamine amidotransferase-like class 1 domain-containing protein 3A, mitochondrial	+	+	+	28

Table S1. List of enriched proteins by LC-MS/MS analysis of ABP MT16-205 labeling
Proteins were enriched under different incubation times (3, 6, 18 h) in HEK293 cells identified by LC-MS/MS analysis of **MT16-205** labeling (130 nM). + shows the protein significantly enriched in each condition. The high-confidence targets found in all treatment conditions are highlighted in grey and UCHL1 is highlighted in green.

Target	Species	Dilution	Catalogue no.	Supplier
ALDH9A1	Rabbit	1:1000	HPA010873	Sigma-Aldrich
BAP1	Mouse	1:500	sc-28383	Santa cruz
HA-tag (C29F4)	Rabbit	1:1000	3724	Cell Signaling Technology
HSP90	Mouse	1:1000	sc-69703	Santa Cruz Biotechnology
UCHL1/PGP9.5	Rabbit	1:2000	14730-1-AP	ProteinTech
UCHL3	Mouse	1:1000	sc-100340	SantaCruz
UCHL5/UCH37	Rabbit	1:1000	ab124931	Abcam
β-ACTIN	Rabbit	1:5000	Ab8227	Abcam
Mouse IgG, HRP	Mouse	1:10000	R-05071-500	Advansta
Rabbit IgG, HRP	Rabbit	1:10000	R-05072-500	Advansta

Table S2. Primary and secondary antibodies used.
Primary and secondary antibodies were used at indicated concentrations for immunoblotting as described in Materials and Methods.

Materials and Methods

Biological and Biochemical Methods and Proteomics

Reagents

Dulbecco's Modified Eagle's Medium (DMEM) high glucose (#41965), fetal bovine serum (FBS, #10500) and penicillin/streptomycin (P/S, #15070) were obtained from Gibco™. DMEM low glucose (#D6046) was obtained from Sigma-Aldrich. Human embryonic kidney 293 cell line (HEK293), colorectal carcinoma cell line (HCT116), a breast carcinoma cell line (Cal51) were obtained from ATCC and verified by STR by The Francis Crick Cell Services. UCHL1 expression in pGEX-6P-1 vector (GST-tagged UCHL1) was a generous gift from Dr C. Das (Purdue University, USA). Recombinant UCHL1 was produced in-house as previously described.[1] Other recombinant DUBs were purchased from Boston Biochem or Ubiquigent. Sequencing grade modified trypsin was obtained from Promega (#V5111). Ultrapure water was obtained on a MilliQ Millipore water purification system. The synthesis of capture reagents AzTB (azido-TAMRA-biotin), and AzRB (azido-arginine-biotin) have been described previously.[2] Compounds **MT16-001** and **MT16-009** were synthesized based on the original report[3] while the synthesis of compound **MT16-205** was modified as described in chemical synthesis. All compounds were dissolved in DMSO and stored at -20 °C.

Biochemical enzymatic assay

DUB enzymatic assay was performed as previously reported.[1] Briefly, varying concentrations of compound were added to a black 384-well plate. Enzymes were diluted in reaction buffer A (20 mM Tris, pH 7.5, 100 mM NaCl, 0.05% Tween-20, 0.5 mg/mL BSA, 5 mM BME) or B (40 mM Tris, pH 7.5, 0.005% Tween-20, 0.5 mg/mL BSA, 5 mM BME), and the diluted enzymes were added to the compound. Enzyme and compound were incubated for 30 min at room temperature. Reactions were initiated by the addition of substrate as previously reported.[1] Reactions were read immediately after substrate addition and following a 2 h incubation at room temperature using a Pherastar Plus (BMG Labtech; λ_{Ex} 540 nm, λ_{Em} 590 nm). IC_{50} values were determined by plotting percentage UCHL1 activity against compound concentration and fitting a four-parameter dose-response curve (Graphpad Prism 5.03).

UCHL1 covalent adduct determination

50 µL of 5 µM recombinant UCHL1 in buffer C (50 mM Tris-HCl, 150 mM NaCl, 1 mM EDTA, 1 mM DTT, pH 7.5) was incubated with 2.5 equiv. of **MT16-001** or **MT16-205** or DMSO for 1 h at room temperature. The excess ligand was removed by washing with buffer C in an Amicon® Ultra MWCO 10 kDa centrifugation spin filter (×3). The protein-adducts were desalted using 3× SDB-XC polystyrene-divinylbenzene copolymer extraction disks, following published methods.[4] Samples were eluted with 50% (v/v) MeCN 0.5% (v/v) formic acid in in LC-MS grade water and analyzed by LC-ESI (ACQUITY UPLC H-Class PLUS instrument (Waters)) using an ACQUITY UPLC BEH C_4 column (Waters) coupled with Waters LCT Premier MS detector.

Cell Culture

Cal51 cells were culture in DMEM high glucose supplemented with 10% FBS and penicillin/streptomycin. HEK293 cells were culture in DMEM low glucose with 10% FBS. All cells were maintained in a humidified incubator at 37 °C and 5% CO_2. Cells were seeded on plates at least 24 h before experiment.

Cellular cytotoxicity assay (EC_{50}) using IncuCyte live-cell image

In triplicate, HEK293 cells (1,000 cells per well) were seeded in 96-well plates (Corning, 3595) and after 24 h, were incubated with 7 serial dilutions of compounds. Cell viability was measured by comparing the occupied area (% confluence) of cell images captured every 2 h for 72 h using an IncuCyte® S3 live-cell time-lapse imaging device (Sartorius). The cell

imaging was analyzed by IncuCyte® analysis software module and the data exported to GraphPad Prism 5.03 for four-parameter dose-response curve fitting and EC_{50} calculation.

Homogeneous time resolved fluorescence (HTRF) assay

HTRF assay was performed as previously reported.1 Briefly, FLAG-UCHL1 stably expressed Cal51 cells were plated overnight prior to incubate with compounds for 1 h at 37 °C. Cells were then washed twice with PBS and lysed with buffer D (50 mM Tris, pH 7.5, 150 mM NaCl, 0.1% NP-40, 0.5% CHAPS, 5 mM $MgCl_2$, 5 mM BME, cOmplete™ EDTA-free protease inhibitor cocktail (Roche) and phosphatase inhibitor (Roche)) and incubated on ice for 30 min. Lysate was combined with Ub-VME probe in a white 384-well plate and incubated for 30 min at room temperature. Detection antibodies, anti-FLAG cryptate antibody (Cisbio #61FG2KLA) and anti HA-XL antibody (Cisbio #610HAXLB) were added to plates. The plates were sealed and incubated overnight at 4 °C then read using a PHERAstar (BMG), and the Delta F and cellular IC_{50} were calculated as previously reported.[1]

In-cell target engagement using HA-Ub-VME

Near-confluent 6-well plate of HEK293 cells were incubated with media containing compounds MT16-001, MT16-205 and MT16-009 at indicated concentrations for 1 h at 37 °C. The cells were washed with PBS and lysed with Tris-lysis buffer (50 mM Tris pH 7.5, 50 mM NaCl, 0.1% (v/v) NP-40, 0.5% (w/v) CHAPS, 2 mM MgCl2, 5 mM BME, 10 U Benzonase, cOmplete™ EDTA-free protease inhibitor cocktail (Roche) and phosphatase inhibitors (Roche)). Lysate (20 µg) were incubated with HA-Ahx-Ahx-Ub-VME (0.5 µg) for 15 min at room temperature. 5× sample loading buffer (160 mM Tris pH 8, 5% (w/v) SDS, 0.025% (w/v) bromophenol blue, 25% (v/v) glycerol) containing 4% (v/v) BME was added and samples heated for 5 min at 95 °C prior to SDS-PAGE and immunoblotting.

Preparation of lysates for ABP MT16-205 labeling and competition

HEK293 cells were seeded overnight prior to incubate with media containing indicated concentration of **MT16-205** or vehicle control (DMSO) for indicated times. For competition experiments, HEK293 cells were pre-incubated with indicated concentrations of parent compounds (**MT16-001**), inactive compounds (**MT16-009**) or DMSO control for 1 h before incubation with ABP **MT16-205**. In both cases, treated cells were washed with PBS (×2) and lysed in PBS-lysis buffer (1% (v/v) Triton X-100, 0.1% (w/v) SDS in PBS supplemented with 1× cOmplete™ EDTA-free protease inhibitor cocktail). The lysate was clarified by centrifugation and the supernatant recovered. The protein concentration was determined using the DC™ protein assay (Bio-Rad) following the manufacturer's protocol. After cell lysis and CuAAC ligation, samples were analyzed by in-gel fluorescence, immunoblotting or proteomics (described below).

Click chemistry (CuAAC) ligation

Lysates were normalized to 1–2 mg/mL in PBS-lysis buffer and incubated with pre-mixed click reagents. The pre-mixed click was prepared separately (6 µL for every 100 µL of lysate)[1, 2]:

- Capture reagent (AzTB for pulldown and in-gel fluorescence or AzRB for proteomics, 10 mM stock in DMSO, 1 vol; final concentration in reaction 100 µM)
- CuSO4 (50 mM stock in water, 2 vol; final concentration in reaction 1 mM)
- TCEP (50 mM stock in water, 2 vol; final concentration in reaction 1 mM)
- TBTA (10 mM stock in DMSO, 1 vol; final concentration in reaction 100 µM)

The pre-mixed click was vortexed and incubated at room temperature for 2 min. The reaction mixture was shaken for 1 h at room temperature. The reaction was quenched with the addition of EDTA (5 mM final concentration). Protein was precipitated by adding MeOH/$CHCl_3$/water at a ratio of 2:0.5:1, relative to the sample volume. The precipitate was washed with cold MeOH, and air-dried for 5 min. The protein pellet was first resuspended in 2% (w/v) SDS in PBS to 10 mg/mL and then diluted with PBS to 1 mg/mL.

SDS-PAGE and in-gel fluorescence analysis for ABP labeled lysate
Typically, protein or lysate samples (10-20 μg) were mixed with 4× Laemmli sample loading buffer (250 mM Tris-HCl pH 6.8, 30% (v/v) glycerol, 10% (w/v) SDS, 0.05% (w/v) bromophenol blue) supplemented with 20% v/v β-mercaptoethanol (BME) and boiled for 10 min at 95 °C. For the ABP labeling experiment, the ABP treated lysates were incubated with 4× Laemmli sample loading buffer supplemented with 1.3% v/v BME (final concentration 0.31% v/v BME) for 5 min without boiling. The lysate samples were separated on 12% (w/v) acrylamide Tris-HCl gels using Tris/glycine/SDS running buffer (0.25 M Tris, 0.2 M glycine, 0.1% (w/v) SDS) at 90 V for 30 min followed by 120 V for 1 h. Gel were imaged on a Typhoon imager (GE Healthcare; λ_{Ex} 532 nm, λ_{Em} 575 nm for TAMRA fluorophore; λ_{Ex} 635 nm, λ_{Em} 575 nm for protein MW markers (Precision Plus All Blue Standards, Bio-Rad). For protein loading quantification, gels were stained with Coomassie blue staining solution overnight, rinsed with water and imaged on an ImageQuant™ LAS 4000 imager (GE Healthcare).

Protein enrichment using Streptavidin magnetic beads
Protein samples (150 μg) were ligated to the capture reagent (AzTB), precipitated and resuspended as described previously. An aliquot (10 μg) was kept for total lysate input before pulldown (TL). The samples (100 μg) were incubated with pre-washed Streptavidin Magnetic Beads (New England Biolabs, 30 μL beads per 100 μg protein) for 2 h at room temperature. Beads were washed with 0.2% (w/v) SDS in PBS (0.5 mL, ×3) and eluted by boiling in 1× sample loading buffer (95 °C, 10 min). Proteins were separated by SDS-PAGE and further analyzed by immunoblotting.

Immunoblotting
Gels were briefly washed with deionised water, and the proteins were then transferred to a 0.45 μm nitrocellulose membrane (Amersham™ Protran®, GE Healthcare) using a wet-tank transfer (Bio-RAD) in Tris-Glycine transfer buffer (25 mM Tris, 190 mM glycine and 20% v/v MeOH) for 1 h at 100 V. Membranes were blocked in 3% (w/v) BSA in Tris-buffer (50 mM Tris pH 7.4, 150 mM NaCl) containing 0.1% (v/v) Tween-20 (TBS-T) for 1 h before incubation with primary antibody in 0.3% (w/v) BSA in TBS-T for 1.5 h at room temperature or overnight at 4 °C (Table S2). The membrane was washed three times with TBS-T for 5 min and incubated with the corresponding HRP-conjugated secondary antibody (α-mouse-HRP or α-rabbit-HRP) in 0.3% (w/v) BSA in TBS-T for 1 h at room temperature. After washing with TBS-T (5 mim, ×3), the membrane was incubated with HRP substrate (Luminata Crescendo, Millipore) and the chemiluminescence signal captured with an ImageQuant™ LAS 4000 imager.

Sample preparation for MS-based proteomic analysis (Label Free Quantification, LFQ)

Proteomics samples were prepared in LoBind microcentrifuge tubes (Eppendorf®). All buffer solutions were prepared fresh and filtered with a 0.22 μm syringe filter prior to use.

Protein lysates, 400 μg in biological triplicates (preparation of protein lysates described above), were ligated with AzRB capture reagent *via* CuAAC reaction, followed by protein precipitation and resuspension as previously described.[1, 5] The protein solution in 0.2% SDS/PBS was cleared by centrifugation (13,000 rpm, 10 min, room temperature) and incubated with pre-washed NeutrAvidin Agarose Beads (60 μL beads/1 mg protein) for 2 h at room temperature with gentle shaking. The supernatant was removed after centrifugation (3,000 rpm, 2 min) and the protein-immobilized beads were sequentially washed with 1% (w/v) SDS in PBS (0.5 mL, ×3) and 50 mM HEPES pH 8.0 (0.5 mL, ×3). After the final wash, the beads were resuspended in 50 mM HEPES pH 8.0 (50 μL) before the addition of TCEP (5 mM) and chloroacetamide (CAA, 10 mM) for 10 min at room temperature. The sequence grade trypsin (0.2 μg, Promega) were added to the bead suspension and incubated overnight at 37 °C in the shaker (1000 rpm). The beads were pelleted, and the supernatant was transferred to a new LoBind tube. The beads were washed with 80 μL of 0.1% (v/v) formic acid in ultrapure water for 10 min with gentle shaking. The beads were pelleted, and the supernatants were

combined and desalted using 3× SDB-XC polystyrene-divinylbenzene copolymer extraction disks, following published methods. The samples were then dried in a SpeedVac Concentrator and stored at -80°C. Peptides were re-suspended in 2% (v/v) MeCN and 0.5% (v/v) TFA in LC-MS grade water for analysis by nanoLC-MS/MS on a QExactive Orbitrap mass spectrometer.[5, 6]

Proteomics sample preparation for whole proteomes analysis
Protein lysates (10 µg) were reduced and alkylated with TCEP (5 mM) and CAA (10 mM), respectively, for 45 min at room temperature. Lysates were then precipitated using CHCl3/MeOH/water as described previously. The protein pellets were resuspended in 50 mM HEPES pH 8.0 to 1 mg/mL and digested with trypsin (1 µg:100 µg, trypsin: lysate) at 37 °C overnight with shaking. After digestion, the peptides were labeled with TMT reagents (0.08 µg TMT reagent per peptide sample) for 2 h at room temperature with gentle shaking. The reaction was quenched by addition of hydroxylamine (1 µL, 5% (w/v)) and the labeled peptides combined and dried in SpeedVac Concentrator at 45 °C. The dried TMT-labeled peptides were re-suspended in 150 µL 1% (v/v) TFA in MilliQ water and cleared *via* centrifugation, followed by fractionation using 3× SDB-RPS styrenedivinylbenzene-reverse phase sulfonated disks and separated into three fractions as previously described.[7] The samples were then analyzed by nanoLC-MS/MS.

NanoLC-MS/MS analysis of LFQ samples
Peptides were separated on an EASY-Spray™ Acclaim PepMap C18 column (50 cm × 75 µm inner diameter, Thermo Fisher Scientific) using a 70-min linear gradient separation of 0-100% solvent B (80% (v/v) MeCN supplemented with 0.1% (v/v) formic acid): solvent A (2% (v/v) MeCN supplemented with 0.1% (v/v) formic acid) at a flow rate of 250 nL/min. The liquid chromatography was coupled to a Q Exactive mass spectrometer *via* an easy-spray source (Thermo Fisher Scientific) which operated in data-dependent mode with survey scans acquired at a resolution of 70,000 at *m/z* 200. Scans were acquired from 350 to 1650 *m/z*. Up to 10 of the most abundant isotope patterns with charge +2 or higher from the survey scan were selected with an isolation window of 2.0 *m/z* and fragmented by HCD with normalized collision energy of 25. The maximum ion injection times for the survey scan and the MS/MS scans (acquired with a resolution of 17,500 at *m/z* 200) were 20 and 120 ms, respectively. The ion target value for MS was set to 10^6 and for MS/MS to 10^5, and the intensity threshold was set to 8.3×10^2.

NanoLC-MS/MS analysis of TMT samples
Peptides were separated on an EASY-Spray™ Acclaim PepMap C18 column (50 cm × 75 µm inner diameter, Thermo Fisher Scientific) using a 3-hour linear gradient separation of 0-100% solvent B (80% (v/v) MeCN supplemented with 0.1% (v/v) formic acid): solvent A (2% (v/v) MeCN supplemented with 0.1% (v/v) formic acid) at a flow rate of 250 nL/min. The liquid chromatography was coupled to a Q Exactive mass spectrometer *via* an easy-spray source which operated in data-dependent mode with survey scans acquired at a resolution of 70,000 at *m/z* 200. Scans were acquired from 350 to 1800 *m/z*. Up to 10 of the most abundant isotope patterns with charge +2 or higher from the survey scan were selected with an isolation window of 1.6 *m/z* and fragmented by HCD with normalized collision energy of 25. The maximum ion injection times for the survey scan and the MS/MS scans (acquired with a resolution of 35,000 at *m/z* 200) were 20 and 120 ms, respectively. The ion target value for MS was set to 10^6 and for MS/MS to 10^5, and the intensity threshold was set to 8.3×10^2.

Proteomics data processing in MaxQuant
MS raw files were processed in MaxQuant (version 1.6.4.0) using the built-in Andromeda search engine and searched against the human reference proteome including isoforms (Uniprot, Taxonomy 9606, accessed January 2019).[8] The multiplicity of the MS search was set as 1 for LFQ. Reporter ion MS2-based quantification was used for TMT protein quantification, selecting pre/defined TMT 10-plex labeling on lysine side chains and peptide

N-termini. Cysteine carbamidomethylation was used as a fixed modification while methionine oxidation and N-terminus acetylation were used variable modifications. Trypsin/P was selected as a digestion enzyme, with a maximum of 2 missed cleavages and minimum 7 residues of peptide length allowed. Unique and razor peptides were selected for protein identification and quantification. Other default parameters were used. MaxQuant-processed data was further analyzed using Perseus (version 1.6.2.3) and GraphPad Prism (version 5.03).

Proteomics data analysis in Perseus

Protein groups and the intensity values were analyzed in Perseus (version 1.6.2.3)[9] and filtered against "Only ID by site", "Reverse" and "Potential contaminant".

For **LFQ quantification**, the replicates of each condition were grouped. The intensity values were log2 transformed and filtered to require at least 2 valid values per condition and 2 unique and razor peptides for each protein. A two-sample t-test was performed across all quantified proteins to compare the peptide spectra intensity values of condition 1 (e.g. ABP-treated samples) versus condition 2 (e.g. DMSO control) and to determine the significantly enriched proteins in each condition (n=3, Permutation-based FDR=0.01, S0=1). Fold-changes for each comparison were computed and plotted against calculated p-values in a volcano plot. The protein groups were matched with the known database and further processed in GraphPad Prism 5.03.

For **TMT quantification**, the TMT intensity values were grouped per TMT multiplex set (TMT set - A/B/C) and per condition (TMT channel - 126-131). The values were log2 transformed and filtered to require at least 2 valid values per condition and 2 unique and razor peptides for each protein. The mean values within each row (TMT set) were subtracted followed by subtraction of the median values across TMT channels (conditions). A two-sample t-test was performed across all quantified proteins to compare the TMT intensity values of condition 1 (e.g. compound-treated samples) versus condition 2 (e.g. DMSO control) and to determine the significantly change of proteins in each condition (n=3, Permutation-based FDR=0.01, S0=1). Fold-changes for each comparison were computed and plotted against calculated p-values in a volcano plot. The protein groups were matched with the known database and further processed in GraphPad Prism 5.03.

Mass spectrometry proteomics data have been deposited to the ProteomeXchange Consortium (http://proteomecentral.proteomexchange.org) *via* the PRIDE partner repository[10] with the dataset identifier **PXD025692**.

Chemical Method

General synthetic methods

All chemicals and solvents were obtained from Sigma-Aldrich UK or VWR international Ltd and used without further purification. All anhydrous conditions were performed in oven-dried glassware under an argon or nitrogen atmosphere. Dried solvents were dispensed using Pure SolvTM solvent drying towers (Innovative technology Inc.). For purifications, HPLC-grade solvents (≥ 99% purity) were used as purchased from Sigma-Aldrich Chemical Co. Ltd. or Fisher Scientific UK. For reaction work-up, technical grade solvents (≥ 95% purity) were used as purchased from VWR or Fisher.

Compounds **MT16-001** and **MT16-009** were synthesized based on the original report[3] while the synthetic route of compound **MT16-205** was modified with the following procedure.

Analytical techniques

Thin layer chromatography (TLC) analysis was performed on Merck silica gel 60 F254 aluminium plates for monitoring reaction progresses. Spots were visualized under UV lamp (254 nm) and/or stained with potassium permanganate for UV-inactive compounds, or 2,4-dinitrophenylhydrazine (DNP) for carbonyl compounds. Flash column chromatography was manually performed on Merck silica gel 60 Å, eluting with solvents as stated, under positive air pressure.

^{1}H and ^{13}C NMR spectra were recorded on a 400 MHz Bruker AV NMR spectrometer at 298 K (400 MHz for ^{1}H and 101 MHz for ^{13}C NMR) in chloroform-d ($CDCl_3$, Acros Organics) as internal reference (δ_H = 7.26 ppm and δ_C = 77.16 ppm). Chemical shifts (δ) are reported in part per million (ppm) relative to tetramethylsilane (TMS) as reference where δ_H and δ_C (TMS) = 0.00 ppm and their assignments are shown as multiplicity (s, singlet; d, doublet; dd, doublet of doublets; t, triplet; q, quartet; m, multiplet; br., broad), coupling constant, and number of protons. The coupling constants (J values), quoted in hertz (Hz) and recorded to the nearest 0.1 Hz, are calculated by MestReNova© NMR software. Residual solvent signals were used as an internal standard, and the characteristic solvent peaks were corrected to the data reported in *Organometallics*, **2010**, 29 (9), 2176-2179.[11]

High-resolution mass spectrometry (HRMS) and LC-MS analyzes were performed by Imperial Mass Spectrometry service using a Waters Acquity UPLC i-Class system comprised of a Waters BEH Acquity C18 column (2.1 mm × 50 mm, operating at 0.5 mL/min), a Waters LCT Premier ES-ToF mass spectrometer, and a photodiode array (detecting from 210 to 280 nm). Eluents of MeCN and water (each with 0.1% (v/v) formic acid) were used as linear gradients (% (v/v) MeCN in water) of: 5% to 95% (0 to 3.2 min), 95% to 5% (3.2 to 3.5 min), and 5% (3.5 to 4.0 min). The *m/z* values are reported in Daltons.

Preparative LC-MS of all final compounds was performed on a Waters RP-HPLC system comprised of a Waters 2767 auto-sampler, a Waters 515 HPLC pump, a XBridge C18 column (5 µm, 4.6 mm × 100 mm, operating at 1.2 mL/min), a Waters 3100 ESI mass spectrometer, and a Waters 2998 photodiode array (detecting from 190 to 700 nm). MeCN and water (each with 0.1% (v/v) formic acid) were used as eluent: 0-10 min 50-98% MeCN, 10-12 min 98% MeCN, 12-13 min 98 to 50% MeCN, 13-17 min 50% MeCN. Flow rate: 1.2 mL/min. LCMS purity of all compounds are ≥ 95%.

Chemical synthesis

Scheme S1. Synthetic route of compound MT16-205

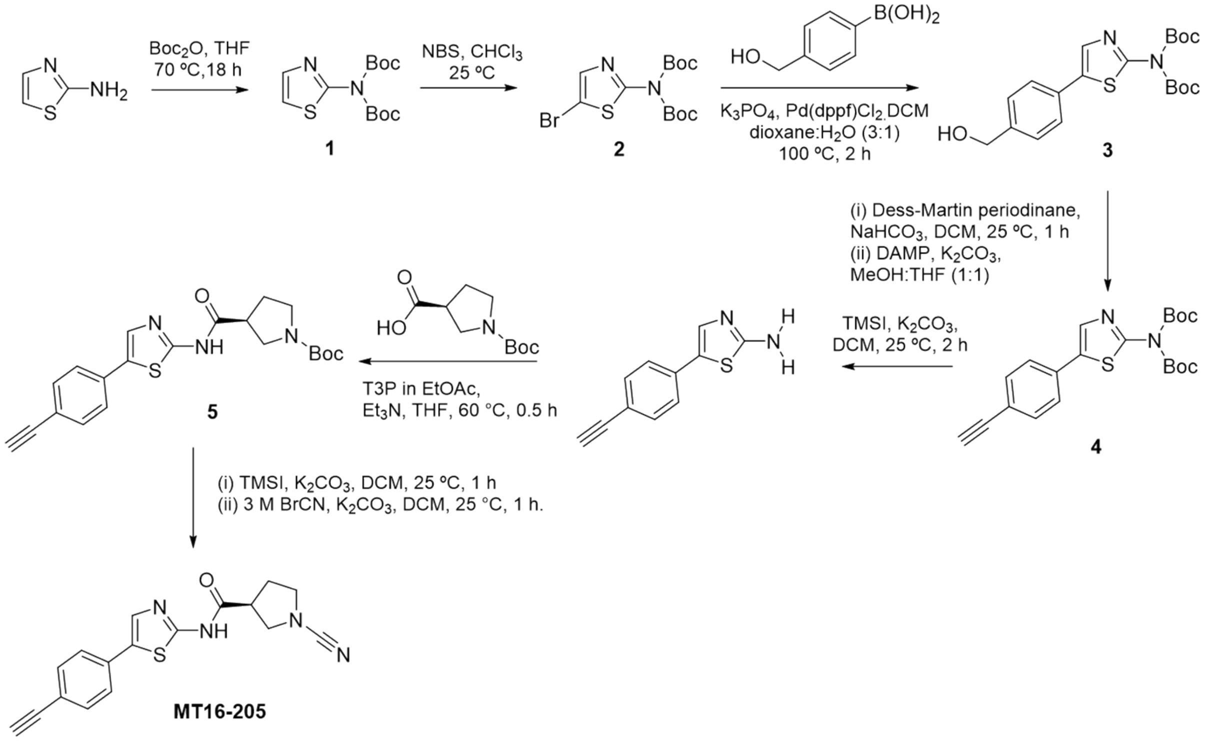

Synthesis of 2-(Di*tert*-butoxycarbonylamino)-1,3-thiazole (1)

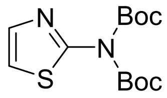

1

To a stirred solution of thiazol-2-amine (1.00 g, 10 mmol) in THF (20 mL) was added Boc_2O (5.30 g, 24 mmol) and DMAP (240 mg, 1.97 mmol). The reaction was stirred at 70 °C for 18 h. Subsequently, the mixture was concentrated *in vacuo* and the resulting solid diluted with CH_2Cl_2 (40 mL) and water (20 mL). The organic layer was separated and the aqueous layer back-extracted with CH_2Cl_2 (2 × 10 mL). The combined organic phases were dried over anhydrous Na_2SO_4, filtered and then concentrated *in vacuo*. The resulting solid was purified by silica column chromatography on a gradient of 0-10% EtOAc/hexane. The required fractions were concentrated *in vacuo* to give a white solid (**compound 1**, 1.97 g, 6.60 mmol, 66%). $\mathbf{R_f}$ = 0.5 (20% EtOAc/hexane); **^{1}H NMR** (400 MHz, $CDCl_3$) δ (ppm): 7.49 (d, *J* = 3.6 Hz, 1H,), 7.12 (d, *J* = 3.6 Hz, 1H), 1.48 (s, 18H). **^{13}C NMR** (101 MHz, $CDCl_3$) δ (ppm): 158.8, 149.8, 139.1, 117.4, 84.6, 27.8; **HRMS** (ES-ToF): *m/z* Calc. for $C_{13}H_{21}N_2O_4S$ $[M+H]^+$: 301.1226, found 301.1222.

Synthesis of 5-Bromo-2-(di*tert*-butoxycarbonylamino)-1,3-thiazole (2)

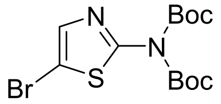

2

To a stirred solution of **compound 1** (1.29 g, 4.30 mmol) in $CHCl_3$ (15 mL) was added NBS (920 mg, 5.20 mmol) portionwise. The reaction was stirred at 25 °C for 2 h. Subsequently, 10% NaOH (aq) solution (20 mL) was added and an extraction carried out with $CHCl_3$ (3 x 20 mL). The combined organic fractions were concentrated *in vacuo* and the resulting oil purified by silica column chromatography on a gradient of 0-20% EtOAc/hexane. The required fractions were concentrated *in vacuo* to give a white solid (**Compound 2**, 1.32 g, 3.51 mmol, 81%). $\mathbf{R_f}$ = 0.6 (20% EtOAc/hexane); **^{1}H NMR** (400 MHz, $CDCl_3$) δ (ppm): 7.39 (s, 1H), 1.53 (s, 18H); **^{13}C NMR** (101 MHz, $CDCl_3$) δ (ppm): 158.7, 149.5, 139.7, 105.8, 85.3, 27.9; **HRMS** (ES-ToF): *m/z* Calc. for $C_{13}H_{20}N_2O_4SBr$ $[M+H]^+$: 379.0337, found 379.0327.

Synthesis of 2-(Di*tert*-butoxycarbonylamino)-5-[*p*-(hydroxymethyl)phenyl]-1,3-thiazole (3)

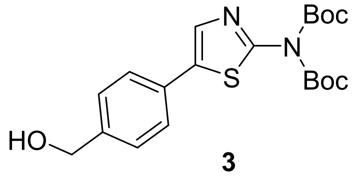

3

A vial was charged with **compound 2** (2.00 g, 5.31 mmol), (4-(hydroxymethyl)phenyl)boronic acid (970 mg, 6.42 mmol), K_3PO_4 (4.40 g, 20.8 mmol), and $Pd(dppf)Cl_2.CH_2Cl_2$ (216 mg, 0.27 mmol). The vial was evacuated and filled with Argon. Subsequently, a N_2 sparged solution of dioxane:water (3:1, 10 mL) was added to the vial and the reaction was stirred at 100 °C for 2 h. The contents were filtered through celite and washed with EtOAc. The filtrate was concentrated *in vacuo* and purified by silica column chromatography on a gradient of 0-40% EtOAc/hexane the product was obtained as a pale brown solid (**Compound 3**, 1.6 g, 4.0 mmol, 75%). **^{1}H NMR** (400 MHz, $CDCl_3$) δ (ppm): 7.64 (s, 1H), 7.50 (d, J = 8.5 Hz, 2H), 7.37 (d, J = 8.5 Hz, 2H), 4.69 (s, 2H), 1.54 (s, 18H); **^{13}C NMR** (101 MHz, $CDCl_3$) δ (ppm): 157.3, 149.7, 141.0, 136.7, 134.3, 130.7, 127.6, 126.4, 84.9, 64.8, 27.8; **HRMS** (ES-ToF): *m/z* Calc. for $C_{20}H_{27}N_2O_5S$ $[M+H]^+$: 407.1641, found 407.1641.

Synthesis of 2-(Di*tert*-butoxycarbonylamino)-5-(*p*-ethynylphenyl)-1,3-thiazole (4)

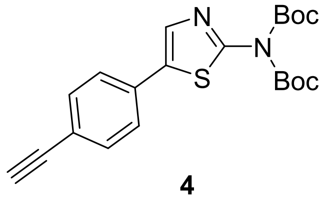

4

To a stirred solution of **compound 3** (740 mg, 1.80 mmol) and $NaHCO_3$ (900 g, 10.7 mmol) in CH_2Cl_2 (10 mL) was added Dess-Martin periodinane (930 mg, 2.20 mmol). The reaction was stirred at 25 °C for 1.5 h. Subsequently, the reaction was quenched with 1:1 $NaHCO_3$:$Na_2S_2O_3$ (aq) (10 mL) and an extraction carried out with CH_2Cl_2 (3 x 15 mL). The combined organic layers were washed with brine solution (25 mL) before being dried over anhydrous Na_2SO_4,

filtered and then concentrated *in vacuo*. The resulting off-white solid was used immediately in the next step without further purification.

To a stirred solution of the resulting off-white solid (740 mg, 1.82 mmol) in 1:1 THF:MeOH (6 mL) was added K_2CO_3 (510 mg, 3.66 mmol). The mixture was stirred for 0.5 h before dimethyl (1-azoacetonyl)phosphonate (330 µL, 2.20 mmol) was added. The reaction was stirred at 25 °C for 18 h. Subsequently, the reaction was quenched with $NaHCO_3$ (aq) solution (10 mL) and an extraction carried out with EtOAc (3 x 10 mL). The combined organic layers were washed with brine solution (25 mL) before being dried over anhydrous Na2SO4, filtered and then concentrated *in vacuo*. The resulting solid was purified by silica column chromatography on a gradient of 0-30% EtOAc/hexane. The required fractions were concentrated *in vacuo* to give a white solid (**Compound 4**, 520 mg, 1.32 mmol, 70%). **^{1}H NMR** (400 MHz, $CDCl_3$) δ (ppm): 7.70 (s, 1H), 7.52 – 7.46 (m, 4H), 3.14 (s, 1H), 1.55 (s, 18H); **^{13}C NMR** (101 MHz, $CDCl_3$) δ (ppm): 158.0, 149.8, 135.8, 135.0, 132.9, 132.0, 126.2, 121.8, 85.1, 83.3, 78.5, 28.0; **HRMS** (ES-ToF): *m/z* Calc. for $C_{21}H_{25}N_2O_4S$ [M+H]$^+$: 401.1545, found 401.1535.

Synthesis of (*S*)-*tert*-Butyl 3-((5-(4-ethynylphenyl)thiazol-2-yl)carbamoyl)pyrrolidine-1-carboxylate (5)

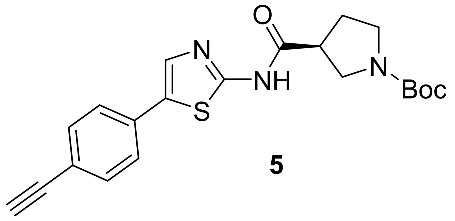

5

Boc deprotection: To a stirred solution of **compound 4** (496 mg, 1.24 mmol) in CH_2Cl_2 (10 mL) was added K_2CO_3 (1.71 g, 12.4 mmol) under Argon. TMSI (400 µL, 2.81 mmol) was added and the reaction stirred at 25 °C with reaction monitoring by LCMS. Subsequently, $NaHCO_3$ (aq) solution was added and an extraction carried out with CH_2Cl_2. The combined organic layers were washed with brine solution, dried over anhydrous Na_2SO_4, filtered and then concentrated *in vacuo*. The resulting crude product was used in the next step without further purification.

Amide Coupling: To a stirred solution of the resulting amine (248 mg, 1.24 mmol) in THF (4 mL) was added to (*S*)-1-(*tert*-butoxycarbonyl)pyrrolidine-3-carboxylic acid (293 mg, 1.33 mmol) and Et_3N (530 µL , 3.50 mmol). The reaction was stirred for 5 min at 25 °C before 50% T3P in EtOAc (2.23 mL, 3.72 mmol) was added. The reaction was stirred at 60 °C for 30 min. Subsequently, distilled water was added and an extraction carried out with EtOAc. The combined organic layers were washed with saturated $NaHCO_3$ (aq) solution, brine solution, dried over anhydrous Na_2SO_4, filtered and then concentrated *in vacuo*. The resulting crude was purified using a 30 g C18 silica column on a gradient of 0-85% MeCN/water (0.1% formic acid modifier). The required fractions were combined and concentrated *in vacuo* to give a white solid (**Compound 5**, 270 mg, 0.68 mmol, 55%). **^{1}H NMR** (400 MHz, $CDCl_3$) δ (ppm): 11.96 (s, 1H), 7.58 (s,1H), 7.55 - 7.47 (m, 4H), 3.89 – 3.54 (m, 4H), 3.46 (m, 1H), 3.24 (m, 1H), 3.16 (s, 1H,), 1.47 (s, 9H); **^{13}C NMR** (101 MHz, $CDCl_3$) δ (ppm): 170.9, 159.1, 154.4, 133.0, 132.2, 131.7, 126.1, 121.9, 83.3, 79.9, 78.7, 48.5, 45.6, 44.8, 43.8, 28.6; **HRMS** (ES-ToF): *m/z* Calc. for $C_{21}H_{24}N_3O_3S$ [M+H]$^+$: 398.1538, found 398.1541.

Synthesis of (*S*)-1-Cyano-*N*-(5-(4-ethynylphenyl)thiazol-2-yl)pyrrolidine-3-carboxamide (MT16-205)

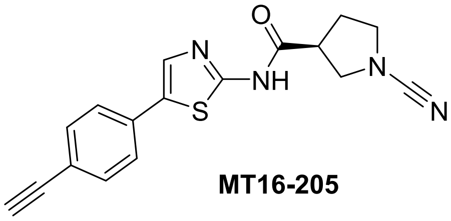

MT16-205

Boc deprotection: To a stirred solution of **compound 5** (270 mg, 0.68 mmol) in CH_2Cl_2 (6 mL) was added K_2CO_3 (938 mg, 6.8 mmol) under Argon. TMSI (200 μL,1.36 mmol) was added and the reaction stirred at 25 °C with reaction monitoring by LCMS. Subsequently, $NaHCO_3$ (aq) solution was added and an extraction carried out with CH_2Cl_2. The combined organic layers were washed with brine solution, dried over anhydrous Na_2SO_4, filtered and then concentrated *in vacuo*. The resulting crude product was used in the next step without further purification.

To a stirred solution of the resulting crude product (270 mg, 0.68 mmol) in CH_2Cl_2 (5 mL) was added K_2CO_3 (470 mg, 3.41 mmol) and 3 M BrCN in CH_2Cl_2 (0.25 mL, 0.75 mmol). The reaction was stirred at 25 °C for 18 h. Subsequently, the solution was diluted with EtOAc (20 mL) and washed with saturated $NaHCO_3$ (aq) solution (2 x 10 mL). The organic phase was separated, dried over anhydrous Na_2SO_4, filtered and then concentrated *in vacuo*. The resulting solid was loaded in CH_2Cl_2 on to a 30 g SNAP silica column and purified on a gradient of 0-2% MeOH/CH_2Cl_2. The required fractions were combined and concentrated *in vacuo* to afford a yellow solid (**Compound MT16-205**, 93 mg, 0.29 mmol, 43%). **^{1}H NMR** (400 MHz, DMSO-d_6) δ (ppm): 12.45 (s, 1H), 7.96 (s, 1H), 7.62 (d, *J*= 8.3 Hz, 2H), 7.50 (d, *J* = 8.3 Hz, 2H), 4.25 (s, 1H), 3.66 – 3.59 (m, 1H), 3.59 – 3.52 (m, 1H), 3.50 – 3.34 (m, 3H), 2.26 – 2.02 (m, 2H); **^{13}C NMR** (101 MHz, DMSO-d_6) δ (ppm): 170.7, 157.3, 135.0, 132.4, 132.0, 130.4, 125.7, 120.5, 117.0, 83.2, 81.6, 52.1, 49.9, 43.1, 29.2; **HRMS** (ES-ToF): *m/z* Calc. for $C_{17}H_{15}N_4OS$ $[M+H]^+$: 323.0967, found 323.0964.

Characterization of compound MT16-001 and MT16-009

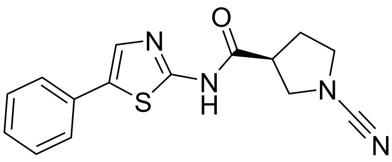

MT16-001

(*S*)-1-cyano-*N*-(5-phenylthiazol-2-yl)pyrrolidine-3-carboxamide (MT16-001)
Compound **MT16-001** (75 mg, 0.25 mmol, 43%). **^{1}H NMR** (400 MHz, DMSO-d_6) δ (ppm): 12.39 (s, 1H), 7.88 (s, 1H), 7.61 (d, J = 7.0 Hz, 2H), 7.43 – 7.39 (m, 2H), 7.34 – 7.27 (m, 1H), 3.67 – 3.59 (m, 1H), 3.58 – 3.52 (m, 1H), 3.51 – 3.41 (m, 2H), 3.40 – 3.32 (m, 1H), 2.20 (m, 1H), 2.25 – 2.03 (m, 1H); **^{13}C NMR** (101 MHz, DMSO-d_6) δ (ppm): 170.6, 156.8, 133.9, 131.5, 131.3, 129.1, 127.5, 125.6, 117.0, 52.2, 49.9, 43.0, 40.4, 29.2.; **HRMS** (ES-ToF): *m/z* Calc. for $C_{15}H_{15}N_4OS$ [M+H]$^+$: 299.0967, found 299.0981.

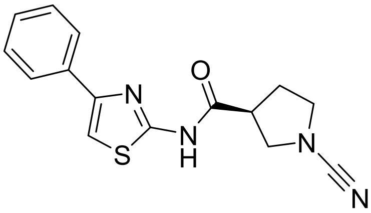

MT16-009

(*S*)-1-cyano-*N*-(4-phenylthiazol-2-yl)pyrrolidine-3-carboxamide (MT16-009)
Compound **MT16-009** (57 mg, 0.19 mmol, 67%). **^{1}H NMR** (400 MHz, DMSO) δ 12.49 (s, 1H), 7.95 – 7.86 (m, 2H), 7.66 (s, 1H), 7.49 – 7.41 (m, 2H), 7.37 – 7.30 (m, 1H), 3.64 (dd, *J* = 9.6, 7.7 Hz, 1H), 3.56 (dd, *J* = 9.6, 6.0 Hz, 1H), 3.51 – 3.40 (m, 2H), 2.27 – 2.16 (m, 1H), 2.15 – 2.04 (m, 1H); **LC-MS** (ES+): *m/z* Calc. for $C_{15}H_{15}N_4OS$ [M+H]$^+$: 299.0967, found 299.15.

References

1. N. Panyain, A. Godinat, T. Lanyon-Hogg, S. Lachiondo-Ortega, E. J. Will, C. Soudy, M. Mondal, K. Mason, S. Elkhalifa, L. M. Smith, J. A. Harrigan and E. W. Tate, *J Am Chem Soc*, 2020, **142**, 12020-12026.
2. M. Broncel, R. A. Serwa, P. Ciepla, E. Krause, M. J. Dallman, A. I. Magee and E. W. Tate, *Angew Chem Int Ed Engl*, 2015, **54**, 5948-5951.
3. WO2016046530 (A1), 2016.
4. J. Rappsilber, Y. Ishihama and M. Mann, *Anal Chem*, 2003, **75**, 663-670.
5. E. M. Storck, J. Morales-Sanfrutos, R. A. Serwa, N. Panyain, T. Lanyon-Hogg, T. Tolmachova, L. N. Ventimiglia, J. Martin-Serrano, M. C. Seabra, B. Wojciak-Stothard and E. W. Tate, *Nat Chem*, 2019, **11**, 552-561.
6. A. Goya Grocin, R. A. Serwa, J. Morales Sanfrutos, M. Ritzefeld and E. W. Tate, *Mol Cell Proteomics*, 2019, **18**, 115-126.
7. M. Hernandez-Valladares, E. Aasebo, O. Mjaavatten, M. Vaudel, O. Bruserud, F. Berven and F. Selheim, *Biol Proced Online*, 2016, **18**, 13.
8. J. Cox, M. Y. Hein, C. A. Luber, I. Paron, N. Nagaraj and M. Mann, *Mol Cell Proteomics*, 2014, **13**, 2513-2526.
9. S. Tyanova, T. Temu, P. Sinitcyn, A. Carlson, M. Y. Hein, T. Geiger, M. Mann and J. Cox, *Nat Methods*, 2016, **13**, 731-740.
10. J. A. Vizcaino, E. W. Deutsch, R. Wang, A. Csordas, F. Reisinger, D. Rios, J. A. Dianes, Z. Sun, T. Farrah, N. Bandeira, P. A. Binz, I. Xenarios, M. Eisenacher, G. Mayer, L. Gatto, A. Campos, R. J. Chalkley, H. J. Kraus, J. P. Albar, S. Martinez-Bartolome, R. Apweiler, G. S. Omenn, L. Martens, A. R. Jones and H. Hermjakob, *Nat Biotechnol*, 2014, **32**, 223-226.
11. G. R. Fulmer, A. J. M. Miller, N. H. Sherden, H. E. Gottlieb, A. Nudelman, B. M. Stoltz, J. E. Bercaw and K. I. Goldberg, *Organometallics*, 2010, **29**, 2176-2179.

^{1}H and ^{13}C NMR Spectra

^{1}H-NMR of compound **1** in $CDCl_3$ (400 MHz)

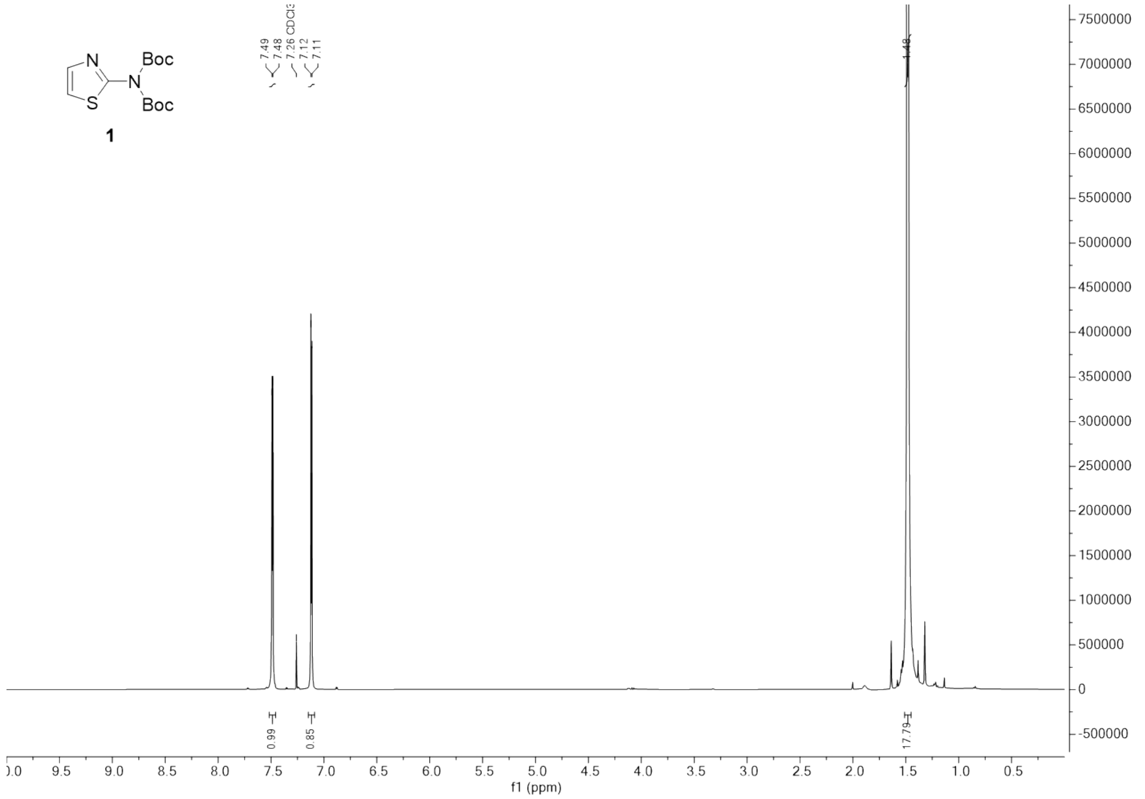

^{13}C-NMR of compound **1** in $CDCl_3$ (101 MHz)

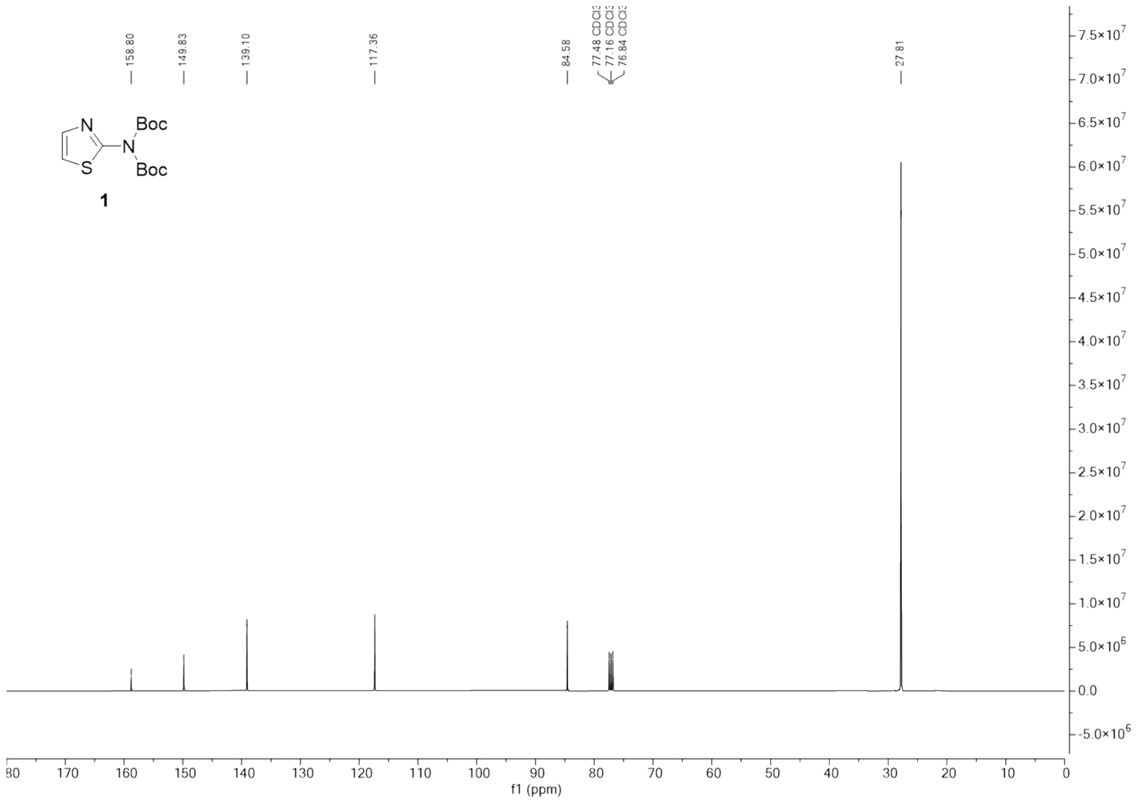

^{1}H-NMR of compound **2** in $CDCl_3$ (400 MHz)

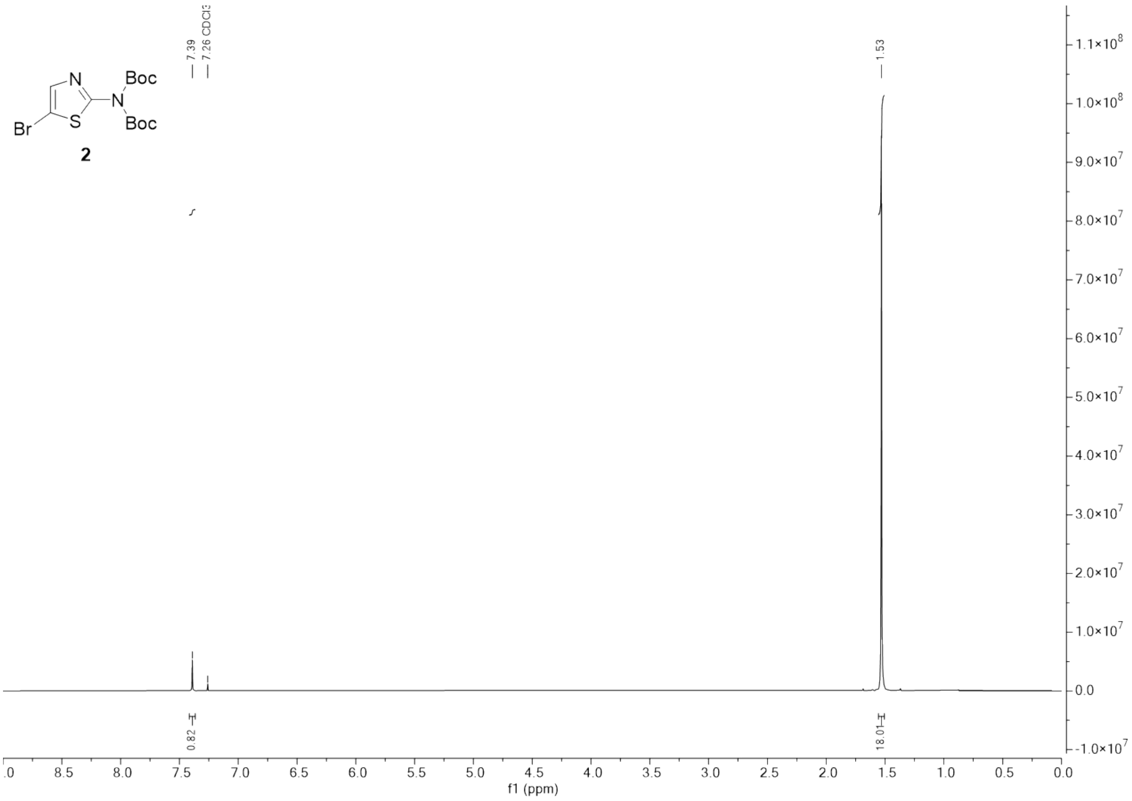

^{13}C-NMR of compound **2** in $CDCl_3$ (101 MHz)

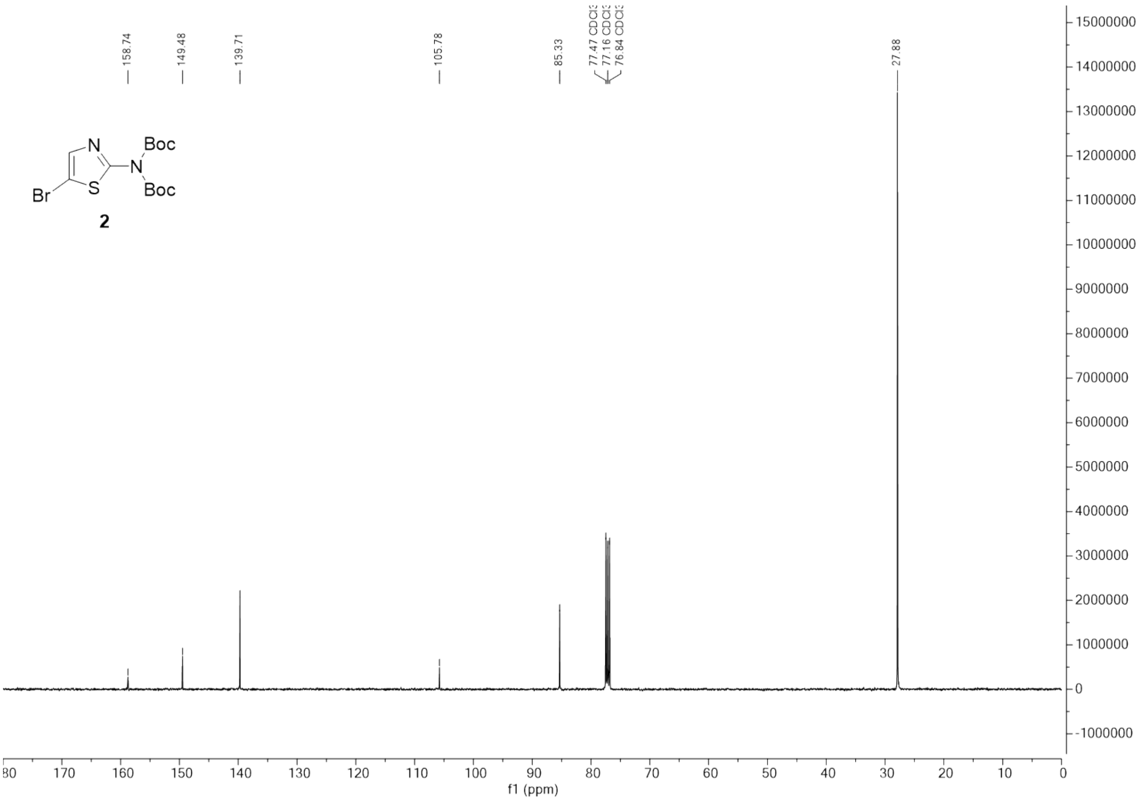

^{1}H-NMR of compound **3** in $CDCl_3$ (400 MHz)

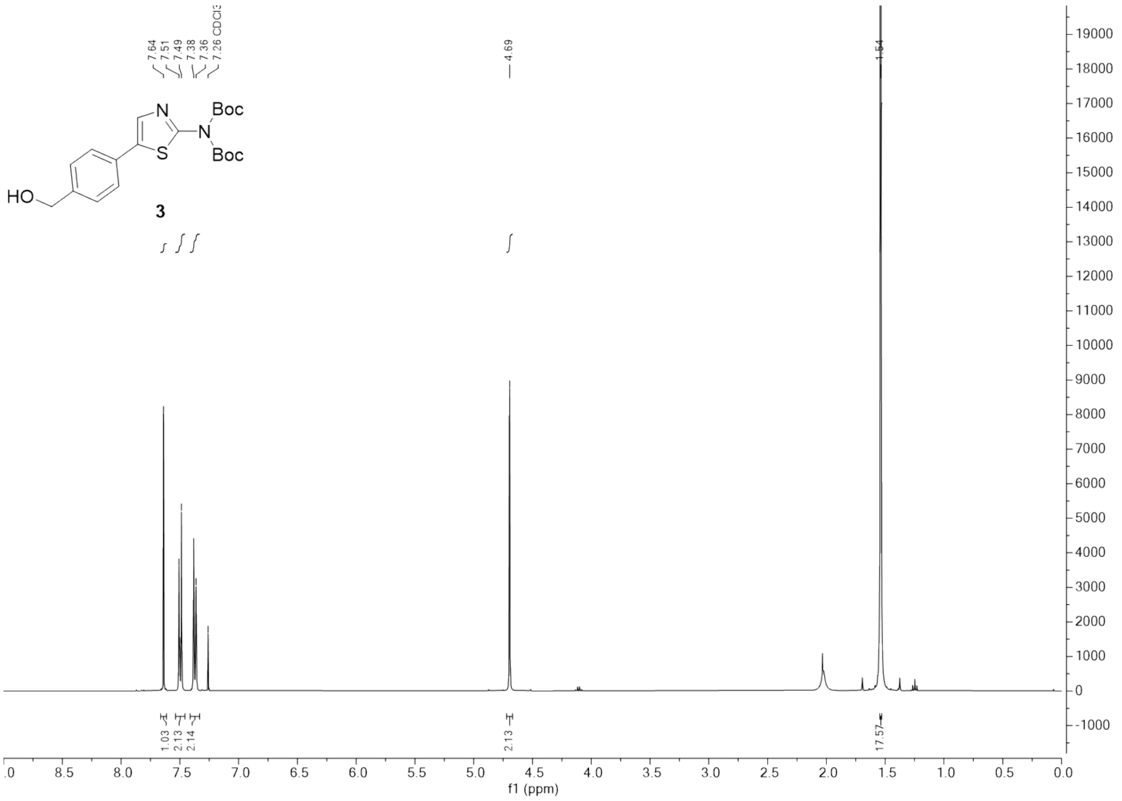

^{13}C-NMR of compound **3** in $CDCl_3$ (101 MHz)

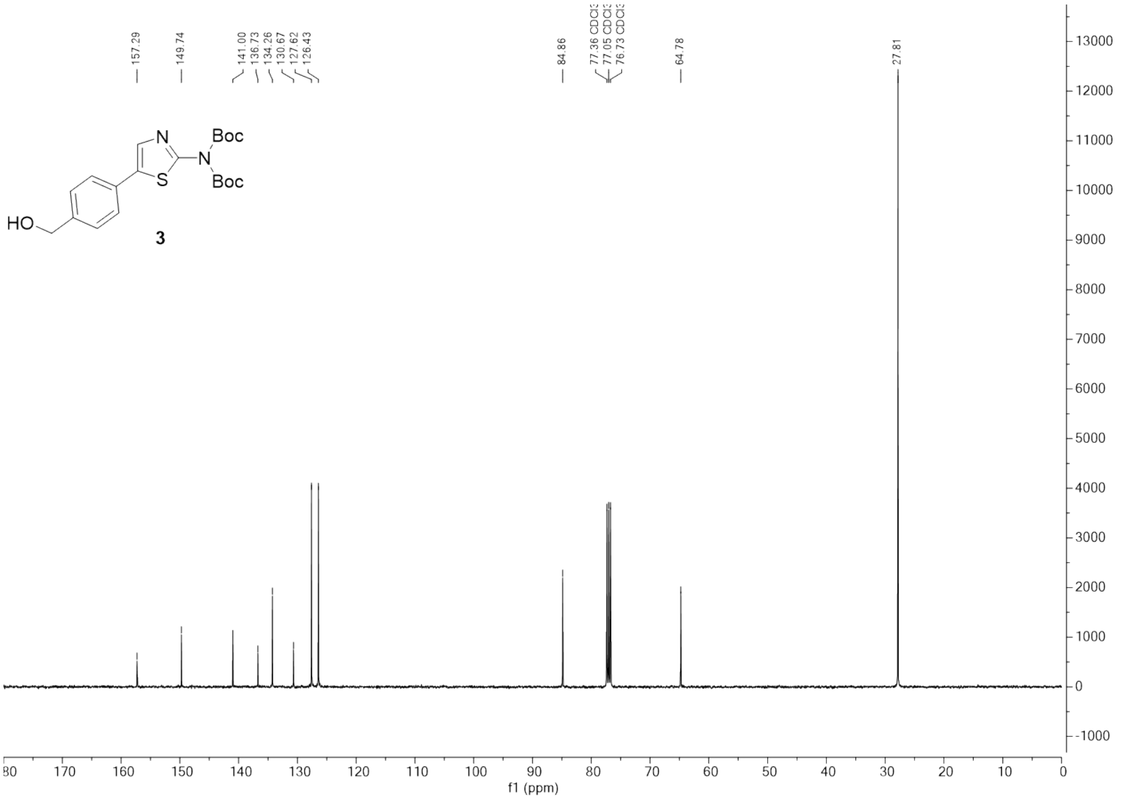

^{1}H-NMR of compound **4** in $CDCl_3$ (400 MHz)

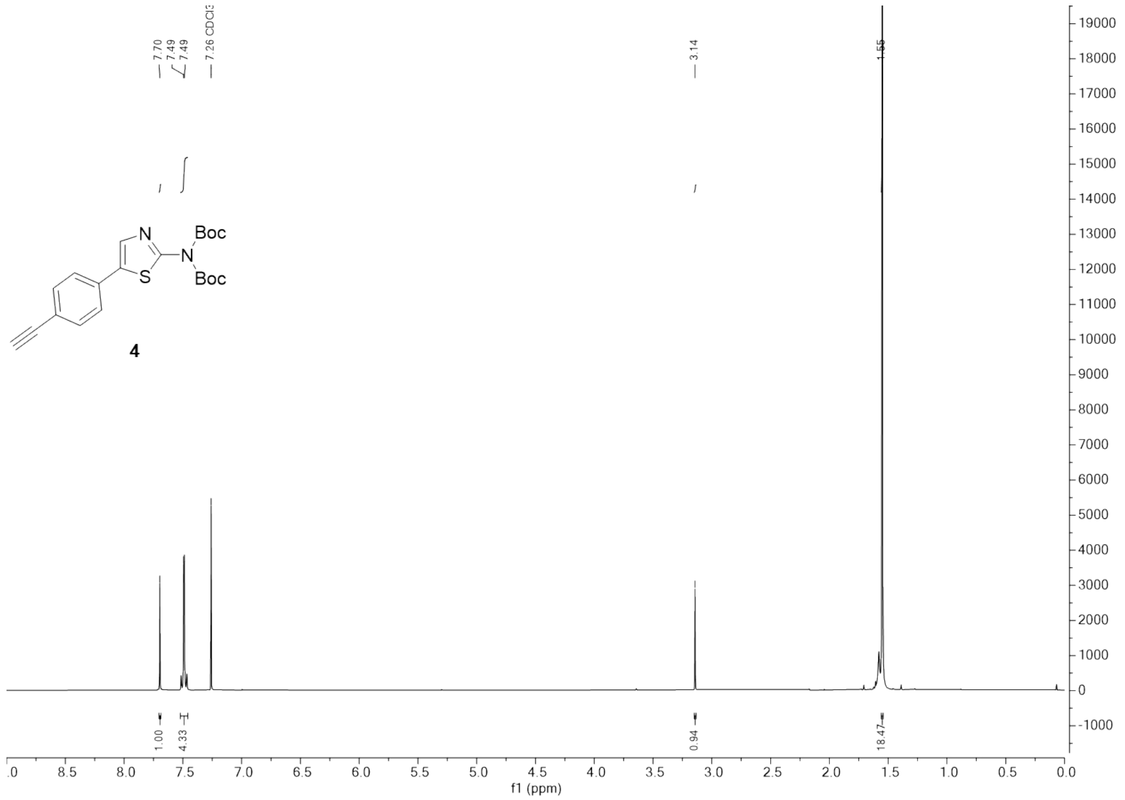

^{13}C-NMR of compound **4** in $CDCl_3$ (101 MHz)

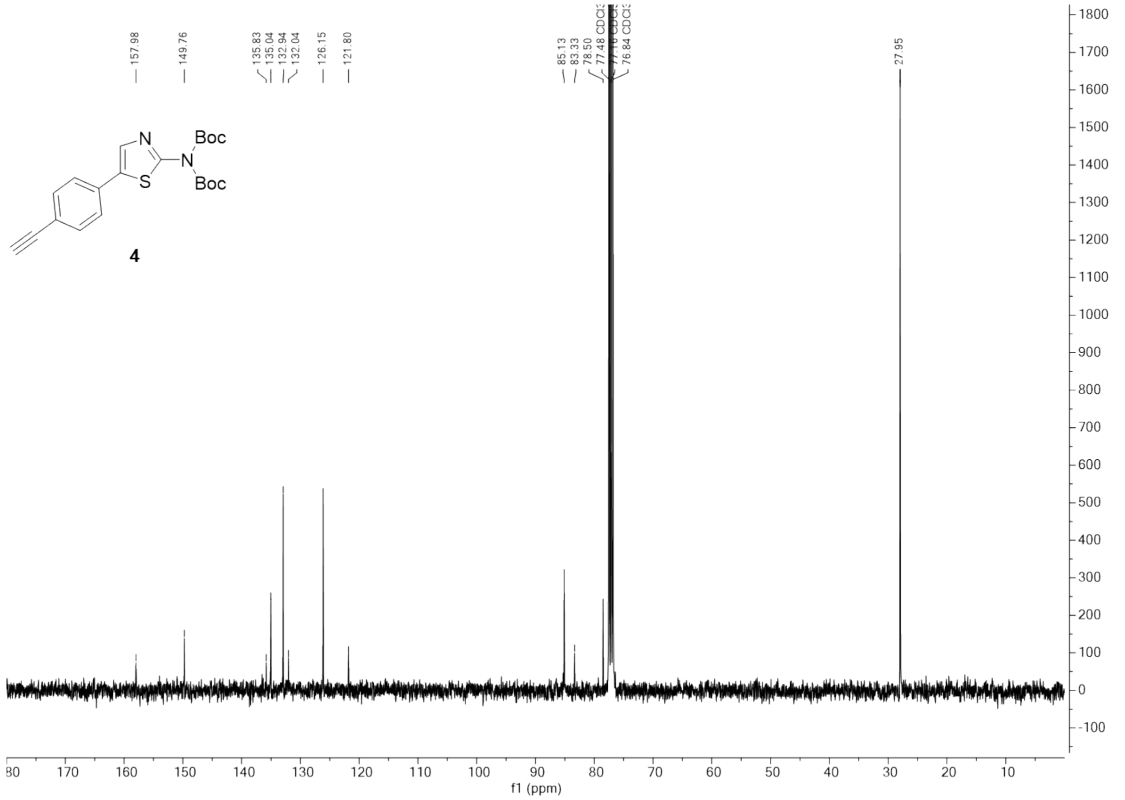

^{1}H-NMR of compound **5** in $CDCl_3$ (400 MHz)

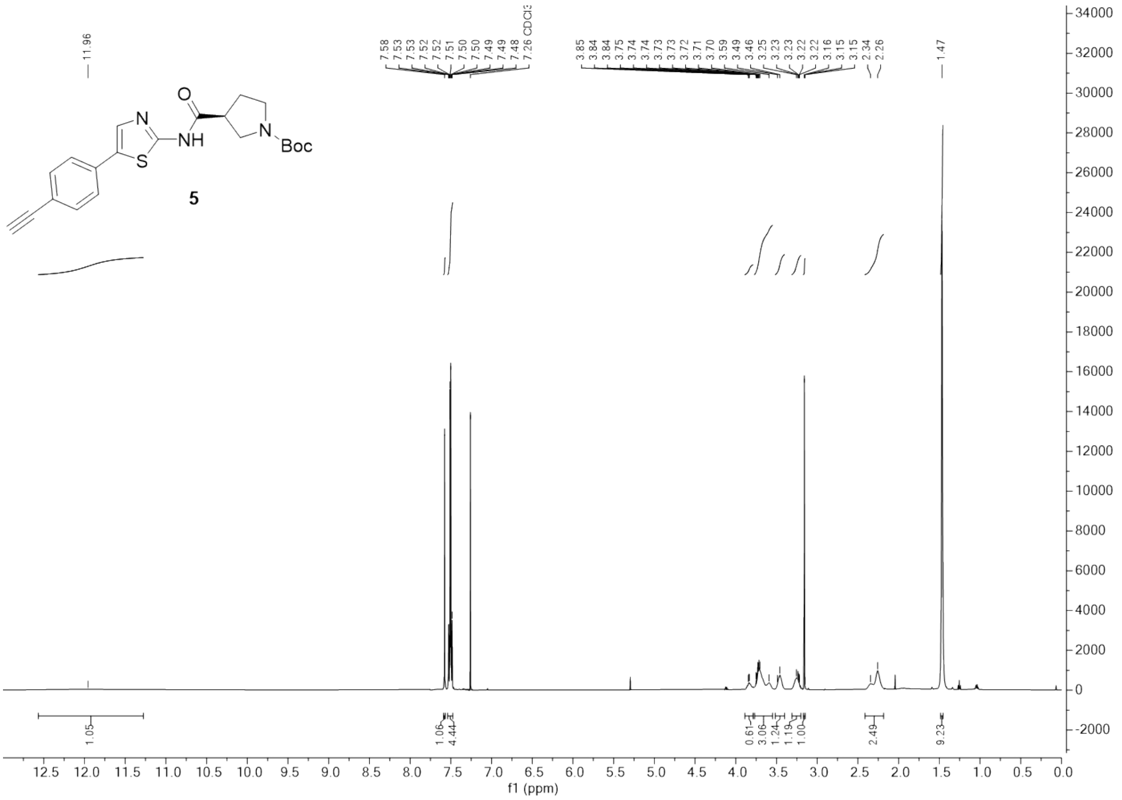

^{13}C-NMR of compound **5** in $CDCl_3$ (101 MHz)

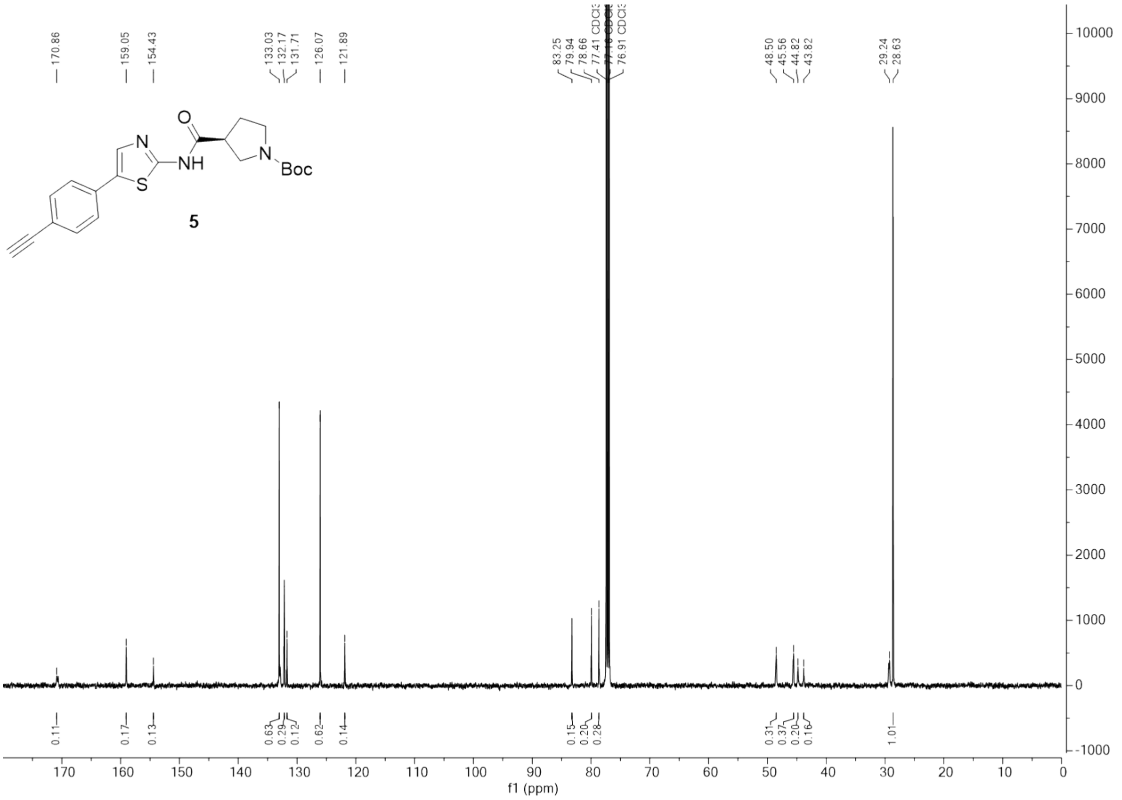

^{1}H-NMR of **MT16-205** in DMSO-d_6 (400 MHz)

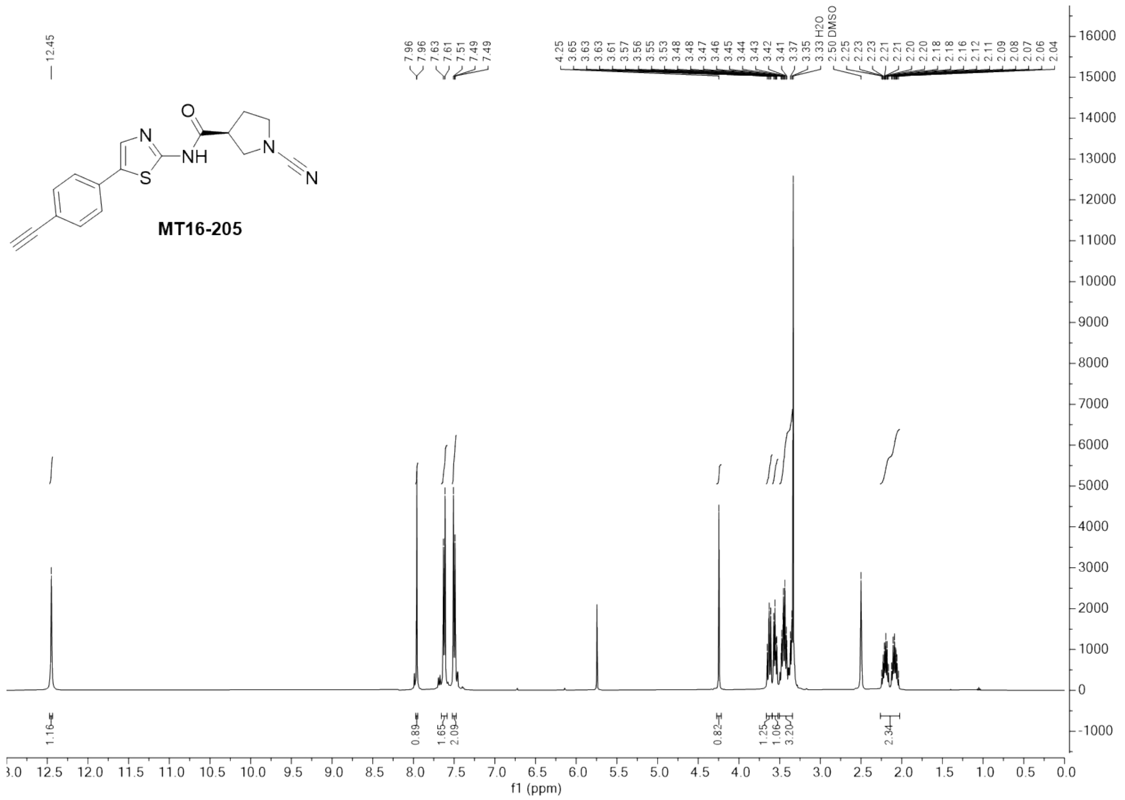

^{13}C-NMR of **MT16-205** in DMSO-d_6 (101 MHz)

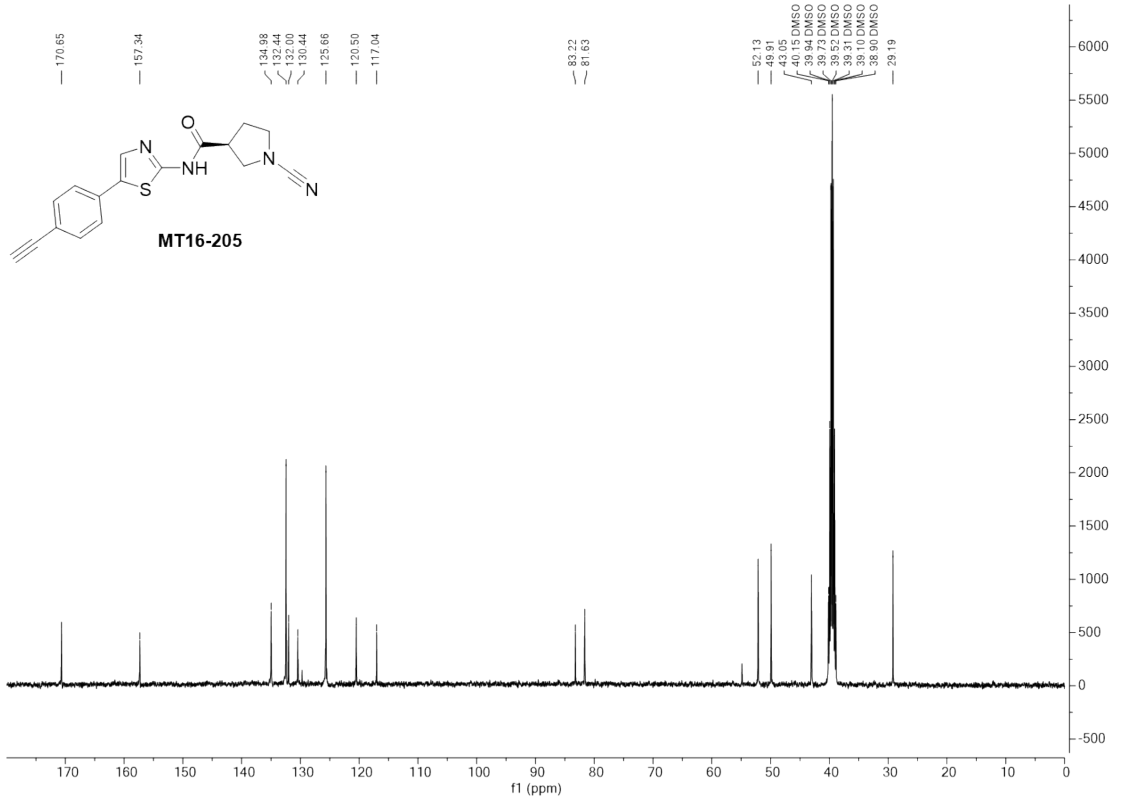

^{1}H-NMR of **MT16-001** in DMSO-d_6 (400 MHz)

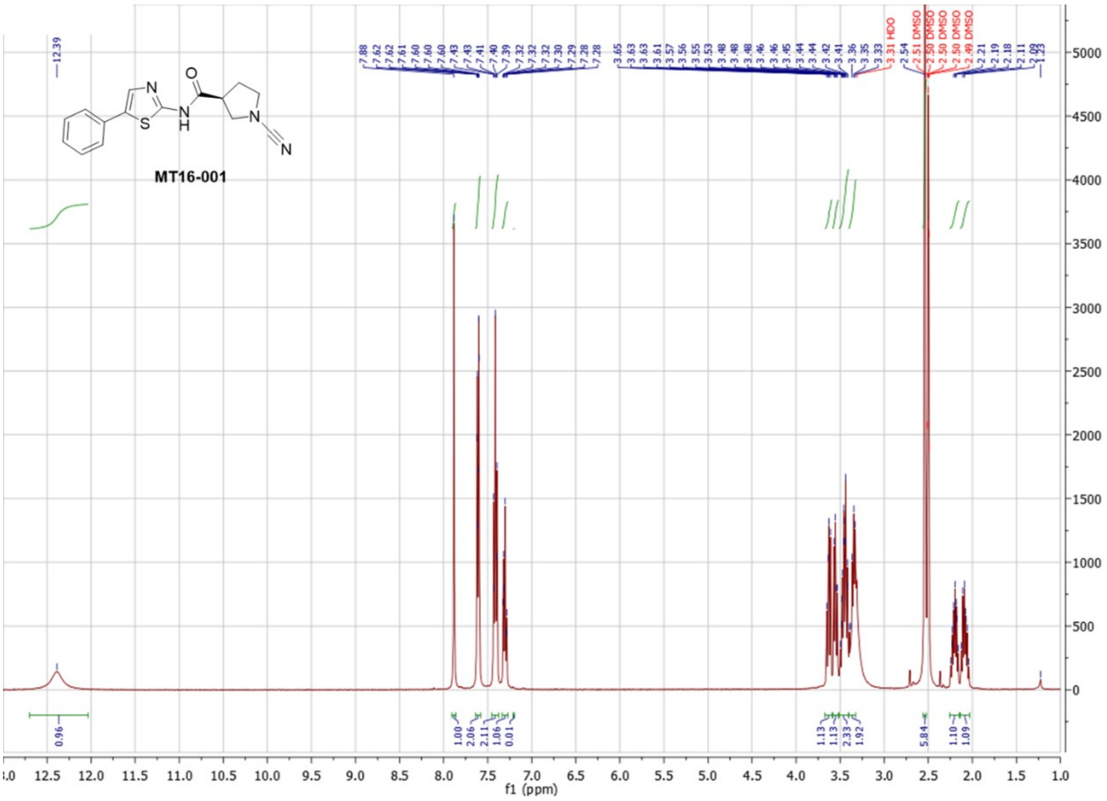

^{13}C-NMR of **MT16-001** in DMSO-d_6 (101 MHz)

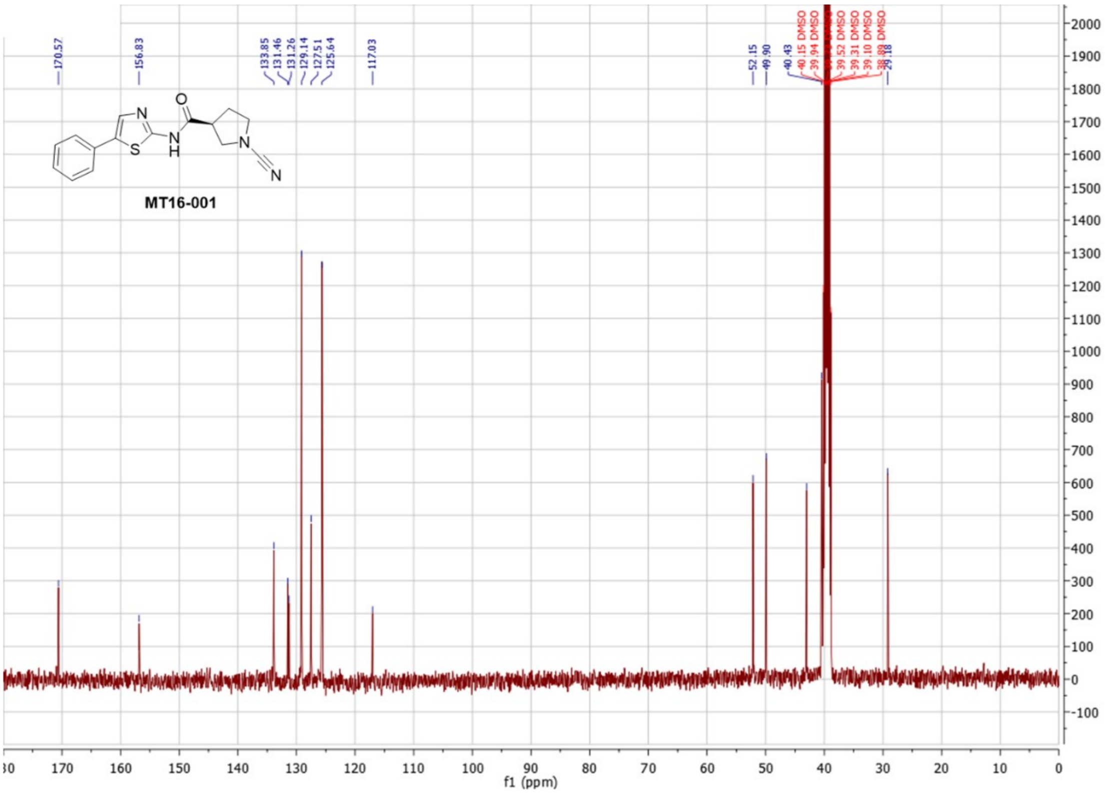

^{1}H-NMR of **MT16-009** in DMSO-d$_6$ (400 MHz)

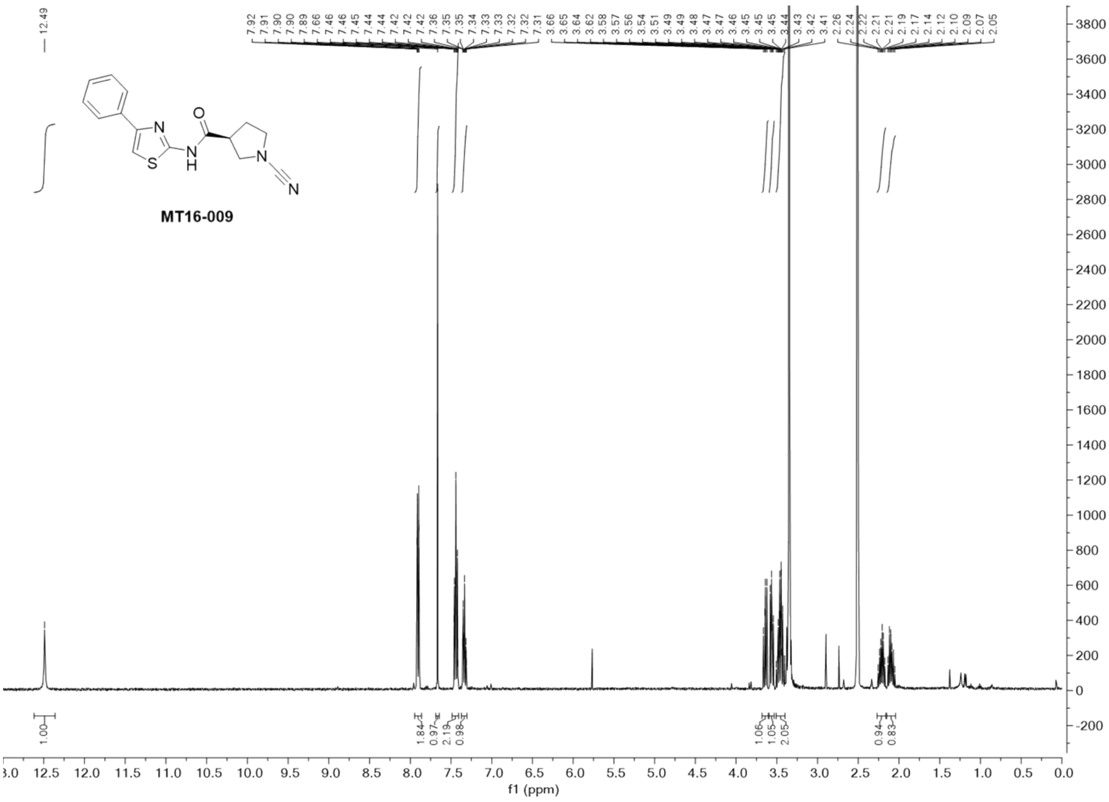

Supplement: MD-012-D1MD00218J-s001 [file MD-012-D1MD00218J-s001.pdf]
